# An innovative colon inflammation-targeting platform for ulcerative colitis treatment

**DOI:** 10.1126/sciadv.aeb1963

**Published:** 2026-10-23

**Authors:** Waliul Islam, Haiming Chen, Ling Li, Kevan J. Salimian, Florin M. Selaru

**Affiliations:** ^1^ Division of Gastroenterology and Hepatology, School of Medicine, Johns Hopkins University, Baltimore, MD 21231, USA.; ^2^ Department of Pathology, School of Medicine, Johns Hopkins University, Baltimore, MD 21224, USA.; ^3^ Department of Oncology, Sidney Kimmel Cancer Center, School of Medicine, Johns Hopkins University, Baltimore, MD 21231, USA.; ^4^ The Institute for Nanobiotechnology, Johns Hopkins University, Baltimore, MD 21231, USA.; ^5^ Department of Biomedical Engineering, School of Medicine, Johns Hopkins University, Baltimore, MD 21231, USA.

## Abstract

The therapeutic options for patients with inflammatory bowel disease (IBD) have recently expanded substantially. Tofacitinib, an oral small-molecule inhibitor of Janus kinase (JAK), recently FDA approved for ulcerative colitis, represented a paradigm shift in a field previously dominated by biologics. Tofacitinib, however, carries a black box warning for major adverse cardiovascular events, an elevated risk of malignancies, risk of blood clots, and pulmonary embolism. In addition, tofacitinib is avoided in pregnancy and used with caution in women of child bearing age due to potential risks to the developing fetus. Notably, these potential complications are all due to the systemic distribution of tofacitinib. To address these issues, we developed Lipo-tm-HA, a liposomal platform incorporating mannitol (m) and hyaluronic acid (HA) for targeting tofacitinib (t) to the inflamed colon. We characterized Lipo-tm-HA and have shown that it (i) remains stable in gastric conditions due to HA coating, (ii) adheres to transferrin in inflamed mucosa due to its negative charge, and (iii) enhances macrophage uptake through mannose receptor–mediated endocytosis. Due to targeting, we show that a single oral administration of Lipo-tm-HA at a dose of 10 milligrams per kilogram is superior in efficacy compared with daily treatment with free tofacitinib in a dextran sodium sulfate–induced colitis model. Collectively, our innovative formulation will enable colonic inflammation targeting, reduce systemic exposure, and allow administration of decreased doses.

## INTRODUCTION

The global prevalence of inflammatory bowel disease (IBD) has risen substantially from 1990, exacerbating the social and economic burden on governments and healthcare systems (*1*, *2*). A 2023 study estimated that nearly 1 in 100 Americans, ∼3.5 million individuals (*2*), suffer with IBD. In 2025, Crohn’s and Colitis Foundation reported that around 10 million people worldwide are living with the illness. One of the major challenges in IBD treatment lies in its heterogeneity and difficulties with tailoring the best treatment for each patient (*3*). Moreover, the high cost of advanced therapies (*4*), potential severe side effects, and lack of curative options further complicate disease management, emphasizing the urgent need for more precise and targeted strategies (*3*). Under these circumstances, pursuing the development of a more effective, safer, and inflammation-targeted drug delivery platform presents a timely, rational, and promising direction for IBD treatment.

Tofacitinib, an orally administered small-molecule inhibitor of Janus kinase (JAK), is approved by the Food and Drug Administration (FDA) for the treatment of ulcerative colitis (UC). The approval of tofacitinib marked a paradigm shift in a treatment landscape historically dominated by biologic therapies (*5*). Tofacitinib is also used either as monotherapy or in combination with other disease-modifying antirheumatic drugs for the treatment of moderate to severely active rheumatoid arthritis, psoriatic arthritis, ankylosing spondylitis, and polyarticular-course juvenile idiopathic arthritis (*6*). Up to 40 to 65% of patients with UC achieve clinical remission with tofacitinib and up to 78% achieve a clinical response (*7*, *8*). Moreover, in those patients who respond to this medication, tofacitinib maintains sustained efficacy over time (*7*, *8*). However, tofacitinib displays several potential complications, such as potential teratogenicity and fetal lethality (*9*), which leads to avoidance of tofacitinib in women of child bearing age. In addition, tofacitinib carries an FDA black box warning due to serious infections, mortality, malignancy major adverse cardiovascular events, and thrombosis secondary to systemic distribution of tofacitinib (*9*–*12*). Here, we report the development of a delivery system of tofacitinib to inflammation sites in the colon in patients with UC, which allows administration of lower doses of tofacitinib and, by extension, minimizes systemic exposure. This delivery platform is composed of a mannitol (m)–engineered liposome coated with hyaluronic acid (HA) for the precise delivery of tofacitinib (t) to inflamed regions of the colon, designating it as Lipo-tm-HA.

Liposomes have been successfully used for drug delivery due to their biocompatibility, dual drug loading capacity, controlled and sustained release, enhanced drug stability, reduced systemic toxicity, and proven regulatory acceptance (*13*, *14*). As of March 2023, the FDA has approved 15 liposomal-formulated nanomedicines for clinical applications, including cancer therapy, antifungal and pulmonary infection treatments, nucleic acid therapies, pain management, viral vaccines, and photodynamic therapy (*15*). While liposomal formulations have been extensively explored in oncology and infectious diseases for targeted drug delivery, however, their clinical application in IBD remains now untested. In this study, we report an oral liposomal JAK inhibitors functionalized with hyaluronic acid and mannitol for targeted delivery to inflamed colonic mucosa and macrophages in UC. The advantages of liposomal drug delivery in our delivery system include (i) codelivery of tofacitinib as an active ingredient and mannitol as a targeting moiety; (ii) sustained drug release; (iii) inflamed colonic mucosa targeting by electrostatic interaction between negatively charged liposomes and positively charged transferrin; (iv) enhanced cell permeability and, by extension, drug availability; and (v) improved therapeutic efficacy. This approach has the potential to address critical clinical unmet needs in UC management and provide therapeutic insights into inflammation-targeted drug delivery.

## RESULTS

### Preparation and characterization

The preparation process of the liposomal formulation with mannitol, tofacitinib, and a hyaluronic acid (HA) coating (Lipo-tm-HA) is illustrated in Fig. 1A. The average hydrodynamic diameter of Lipo-tm-HA was measured at 280 ± 20 nm by dynamic light scattering (DLS), while transmission electron microscopy (TEM) revealed an average particle size of 130 ± 20 nm (Fig. 1, B and C). Meanwhile, we prepared two other formulations: Lipo-t (liposomal-tofacitinib) and Lipo-tm (liposomal tofacitinib with mannitol). In comparison, the hydrodynamic diameters of Lipo-t and Lipo-tm were 170 to 220 nm by DLS (fig. S1A), with corresponding TEM-derived particle sizes of 100 to 120 nm (fig. S1, B and C). The zeta potential of Lipo-t was neutral (−0.02 mV); however, upon mannitol incorporation, it shifted to a slightly negative charge (−18 mV), and, following HA coating, it became even more negative (−32 mV) (Fig. 1D and fig. S1D). The particle size, polydispersity index (PDI), and surface charge of the different liposomal formulations are shown in fig. S1 (E to G).

**Fig. 1.**
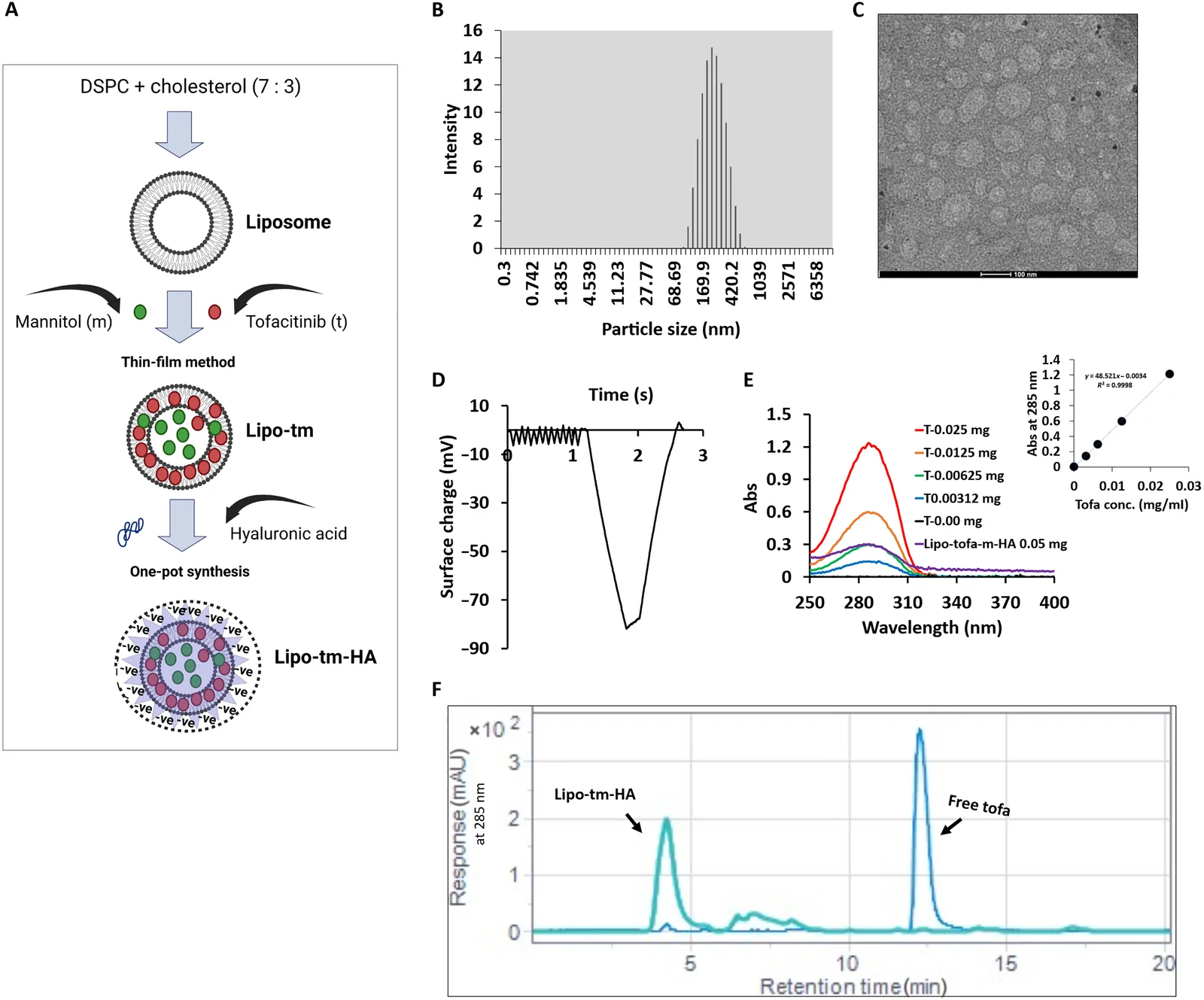
Preparation and characterization of Lipo-tm-HA. (**A**) Schematic illustration of the mannitol-engineered liposomal formulation of tofacitinib (Lipo-tm-HA). DSPC, distearoylphosphatidylcholine. (**B**) The hydrodynamic diameter of Lipo-tm-HA (2 mg/ml in deionized water) was measured by dynamic light scattering (DLS) using cumulant and histogram methods, revealing an average size of 280 ± 20 nm. (**C**) Transmission electron microscopy (TEM) imaging, following staining with 0.01% phosphotungstic acid, showed a smaller particle size of ∼130 ± 20 nm. (**D**) The zeta potential of Lipo-tm-HA (5 mg/ml in deionized water) was −32 mV, indicating a negatively charged surface. (**E**) The tofacitinib loading in the liposomal formulation was quantified by ultraviolet-visible (UV-Vis) spectroscopy using a standard curve of free tofacitinib. The inset shows the linear standard curve at different concentrations, with the maximum absorbance observed at 285 nm. (**F**) Size exclusion chromatography (SEC) of Lipo-tm-HA and free tofacitinib was performed using an Agilent HPLC system with a Shodex GF-310 HQ column (7.5 mm by 300 mm) and photodiode array detection at 285 nm. The black arrows indicate the elution peaks of Lipo-tm-HA and tofacitinib (free tofa). This figure was created and resized in high resolution using BioRender. Selaru, F. (2026), https://BioRender.com/h7qtwb0.

The tofacitinib loading in the liposomal formulation was quantified using ultraviolet-visible (UV-Vis) spectroscopy after removing unbound free drug by dialysis, revealing that Lipo-tm-HA contained ∼10 to 12% (w/w) tofacitinib, based on the standard curve (Fig. 1E). High-performance liquid chromatography (HPLC) with size exclusion chromatography (SEC) confirmed a single elution peak of Lipo-tm-HA at 4 to 5 min, whereas free tofacitinib eluted at 12 to 13 min (Fig. 1F). Additionally, we developed rhodamine-labeled fluorescent liposomes, designated as Lipo-r (liposomal-rhodamine), Lipo-rm (liposomal-rhodamine with mannitol), and Lipo-rm-HA (liposomal-rhodamine with mannitol coated with hyaluronic acid). These formulations contained ∼2 to 3% rhodamine (fig. S2, A and B) and homogenous solution in water (fig. S2C). The DLS analysis showing a single peak corresponding to an average hydrodynamic diameter of 300 ± 30 nm (fig. S2D). The physicochemical properties of the various formulations are summarized in table S1.

### Stability and drug release profile

We designed the tofacitinib platform to resist degradation in the stomach due to low pH. We hypothesized that the incorporation of a hyaluronic acid (HA) coating onto the liposomal surface will enhance stability in the gastric environment following oral administration. The HA coating serves as a protective barrier, maintaining liposome integrity under acidic conditions and subsequently facilitating the dissolution and controlled release of tofacitinib upon transition to the intestinal milieu (Fig. 2A). HA-coated liposomes in Lipo-tm-HA demonstrated exceptional stability in gastric juice, retaining their structural integrity for up to 5 days at 37°C, as evidenced by a single peak in DLS analysis (Fig. 2B). In contrast, upon exposure to intestinal fluid, Lipo-tm-HA exhibited rapid swelling, leading to the emergence of two distinct peaks and aggregation within 30 min, indicating the disassembly of the HA coating (Fig. 2B).

**Fig. 2.**
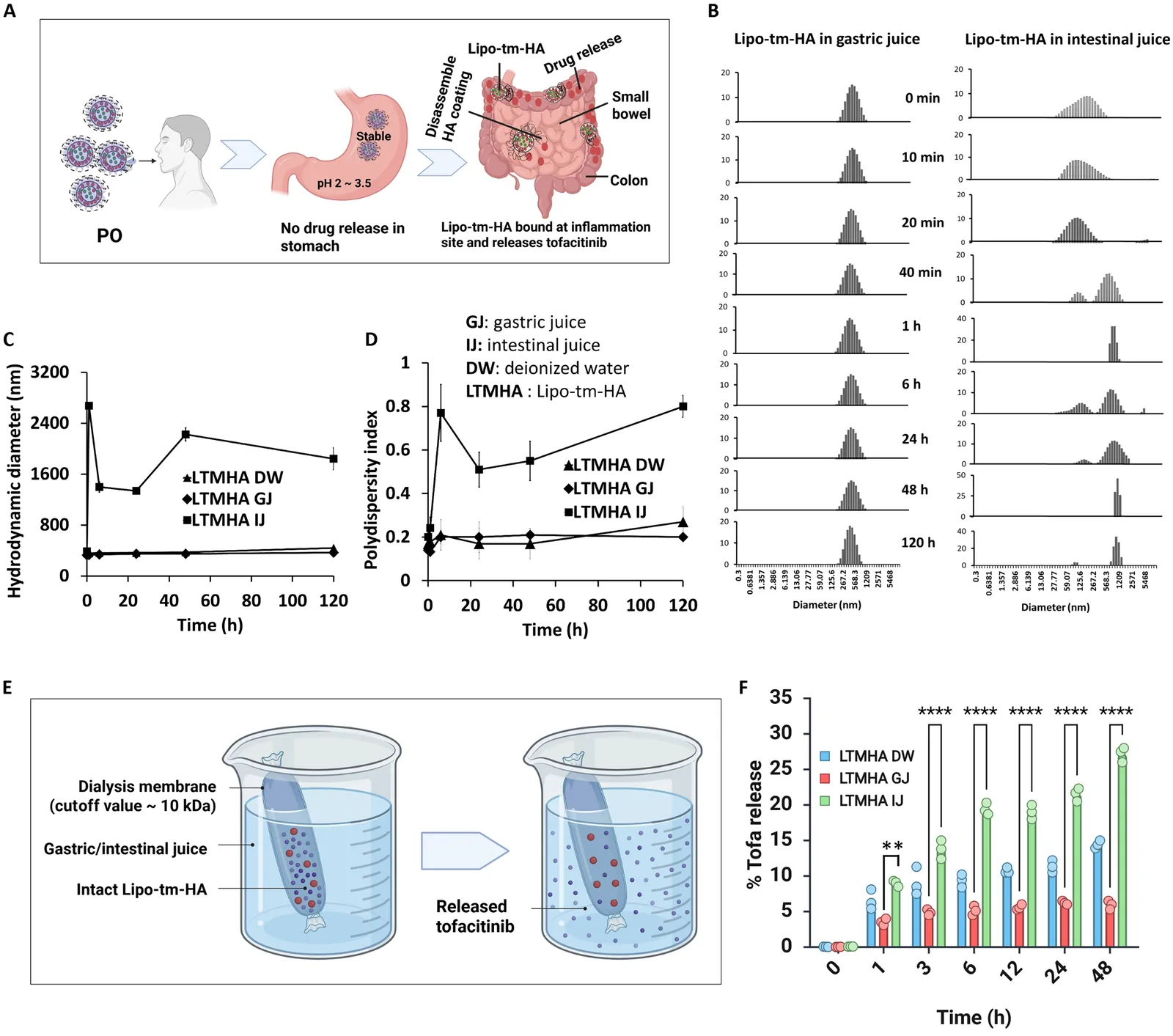
Stability and release profile of Lipo-tm-HA. (**A**) Schematic representation of the proposed mechanism of Lipo-tm-HA accumulation in the inflamed colon following oral administration, bypassing degradation in the gastric environment. (**B** and **C**) Stability assessment of Lipo-tm-HA in simulated gastric and intestinal juices at 37°C, monitored by DLS. (**D**) Changes in PDI of Lipo-tm-HA in gastric juice, intestinal juice, and water. (**E**) Schematic illustration of the drug release study using a Visking tube (MW cutoff: 10 kDa). Tofacitinib encapsulated in Lipo-tm-HA remained within the tube, while the released free drug diffused into the surrounding solution. (**F**) Quantification of released tofacitinib by UV-Vis spectroscopy, with the percentage release calculated relative to the initial drug content in the Lipo-tm-HA formulation. Data are presented as means ± SD (*n* = 3). Statistical significance: **P < 0.01; ****P < 0.0001. LTMHA, Lipo-tm-HA; GJ, gastric juice; IJ, intestinal juice; h, hours. This figure was created and resized in high resolution using BioRender. Selaru, F. (2026), https://BioRender.com/h7qtwb0.

Liposomal formulations lacking the HA coating displayed instability in both gastric and intestinal fluids, with a broader peak and notable aggregation observed within 1 hour (fig. S3). Additionally, Lipo-tm-HA exhibited a broad DLS peak in deionized water after 6 hours of incubation, further highlighting its environmental responsiveness (fig. S4). Furthermore, measurements of hydrodynamic diameter and PDI in both gastric and intestinal fluids revealed that particle size and PDI remained unchanged in gastric juice, whereas significant alterations were observed in intestinal fluid (Fig. 2, C and D). These findings strongly suggest that the HA-coated liposomal formulation remains stable in the acidic gastric environment but undergoes controlled disassembly in the intestine.

Next, we examined the drug release profile of Lipo-tm-HA in both gastric and intestinal fluids, with the experimental workflow depicted in Fig. 2E. As anticipated, the release of tofacitinib from the liposomal formulation was significantly higher in intestinal juice compared with gastric juice (*P* < 0.001, Fig. 2F). Notably, tofacitinib release remained below 10% in gastric fluid even after 48 hours of incubation at 37°C, demonstrating exceptional stability. However, in intestinal juice, drug release increased progressively, reaching 9, 13, 20, 23, and 30% at 1, 3, 6, 12, 24, and 48 hours, respectively (Fig. 2F). Note that tofacitinib release was relatively higher in deionized water than in gastric juice (Fig. 2F), further reinforcing our hypothesis that Lipo-tm-HA remains highly stable in the acidic gastric environment while facilitating efficient drug release in the intestine. Free tofacitinib in dialysis experiments (simulated gastric or intestinal juice) showed complete release (>95%) within 1 to 2 hours (fig. S5), confirming that liposomal sustained release is carrier specific, not medium/dialysis artifacts.

### Transferrin targeting in inflamed colon

During inflammation, the colonic mucosa of affected areas acquires a positive charge due to the accumulation of cationic proteins such as transferrin, antimicrobial peptides, and other positively charged molecules at the compromised epithelial surface (*16*, *17*). This phenomenon arises from the disruption of the protective mucus layer, which ordinarily serves as a barrier, preventing the excessive deposition of these charged biomolecules (*17*). We hypothesize that a negatively charged delivery platform, justifying our choice for a liposomal platform, would increase targeting of transferrin to positively charged inflamed areas of the GI tract (Fig. 3A).

**Fig. 3.**
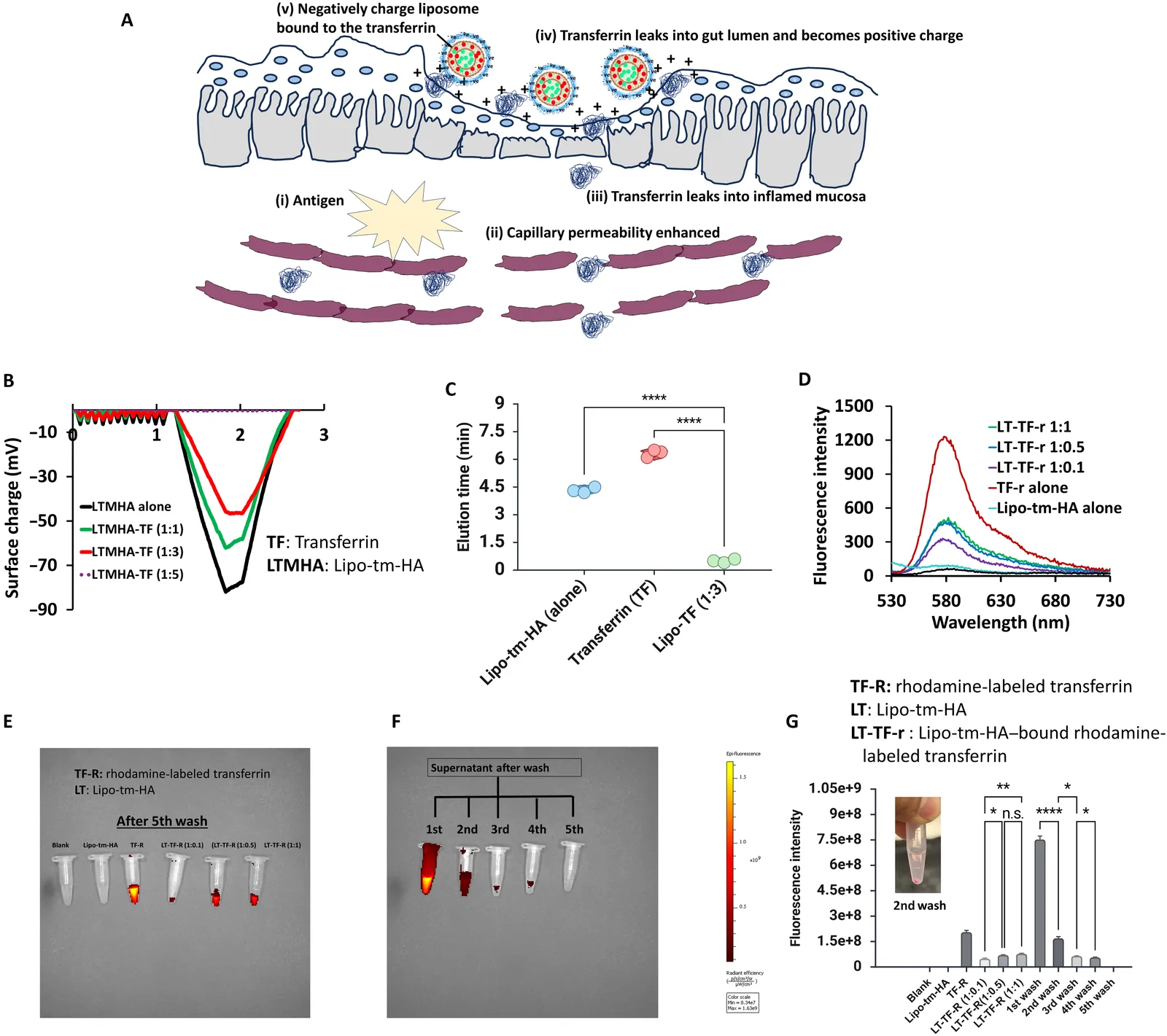
Binding of Lipo-tm-HA to transferrin. (**A**) Schematic illustration depicting transferrin leakage in the inflamed colon, leading to a positively charged microenvironment, and facilitating the electrostatic binding of the negatively charged Lipo-tm-HA. (**B**) Surface charge neutralization of Lipo-tm-HA following coincubation with transferrin for 30 min at 37°C. The bound and unbound transferrin were separated by ultracentrifugation (12,000*g*, 15 min). (**C**) Elution time shift of Lipo-tm-HA after binding with transferrin evaluated by SEC. (**D**) Confirmation of transferrin binding by fluorescence spectroscopy using rhodamine-labeled transferrin (TF-R). (**E**) Fluorescence imaging (IVIS) visualizing Lipo-tm-HA-bound transferrin at different ratios after five times wash. (**F**) Supernatant fluorescence imaging after sequential washing. Following five washing steps, no fluorescence signal was detected in the supernatant, confirming complete removal of unbound transferrin. (**G**) Quantification of transferrin binding at varying Lipo-tm-HA:TF-R ratios. The inset displays the image of the second wash, with transferrin in the supernatant and liposomes in the precipitate. LT-TF-r denoted Lipo-tm-HA–bound rhodamine-labeled transferrin. Data are presented as means ± SD (*n* = 3). Statistical significance: *P < 0.05; **P < 0.01; ****P < 0.0001; n.s., not significant. (A) Rebuilt with permission from Tirosh *et al.* (*37*). Copyright 2009 American Chemical Society (license no. 6190860812302). This figure was created and resized in high resolution using BioRender. Selaru, F. (2026), https://BioRender.com/h7qtwb0.

To test this hypothesis, we first evaluate the binding affinity of transferrin to our negatively charged Lipo-tm-HA. Surface charge analysis following coincubation of transferrin with Lipo-tm-HA, followed by ultracentrifugal separation, revealed a dose-dependent neutralization of the liposomal charge, indicating a successful and effective interaction between Lipo-tm-HA and transferrin (Fig. 3B). To further elucidate the molecular size of Lipo-tm-HA following transferrin binding, we performed SEC in HPLC system. Our analysis revealed the disappearance of the transferrin peak, while the Lipo-tm-HA-transferrin complex eluted within the void volume (fig. S6, A and B), and the elution time of Lipo-tm-HA (4.2 min) and transferrin (6 min) shifted to void volume (0.25 min) (Fig. 3C).

We also substantiate this binding with an ultracentrifugation assay using fluorescently labeled transferrin. Even after the separation of unbound transferrin, a significant fluorescence signal persisted within the Lipo-tm-HA fraction examined by fluorescence spectroscopy (Fig. 3D) and fluorescence imaging (Fig. 3E), confirming successful transferrin association. Notably, after five consecutive washing steps, no detectable fluorescence signal was observed in the supernatant, as determined by fluorescence spectroscopy and imaging (Fig. 3, F and G, and fig. S6C). This indicates the complete removal of unbound transferrin from the solution.

DLS measurements showed no significant changes in the hydrodynamic diameter of Lipo-tm-HA upon transferrin binding (fig. S7A), yet the transferrin peak disappeared after coincubation (fig. S7A). UV-Vis analysis showed no major absorbance shifts, but spectral broadening suggests altered electronic environments from Lipo-tm-HA–transferrin interactions (fig. S7B).

### Macrophage targeting

Macrophages are the most abundant immune cells in the intestinal tract and express high levels of mannose receptors (*18*, *19*). Based on this observation, we hypothesized that a mannitol-derived formulation could target mannose receptors and, by extension, activated macrophages in inflamed regions. To investigate this hypothesis, we examined the dose-dependent cellular uptake of fluorescently labeled mannitol- and nonmannitol-derived liposomal formulations in RAW 264.7 macrophage cells. Notably, our findings revealed that the mannitol-derived liposomal formulation (Lipo-rm) exhibited significantly (*P* < 0.01) higher uptake after 12 hours of incubation at 37°C compared with both the free fluorescent dye rhodamine and the nonmannitol-derived liposome (Lipo-r) (Fig. 4, A and B).

**Fig. 4.**
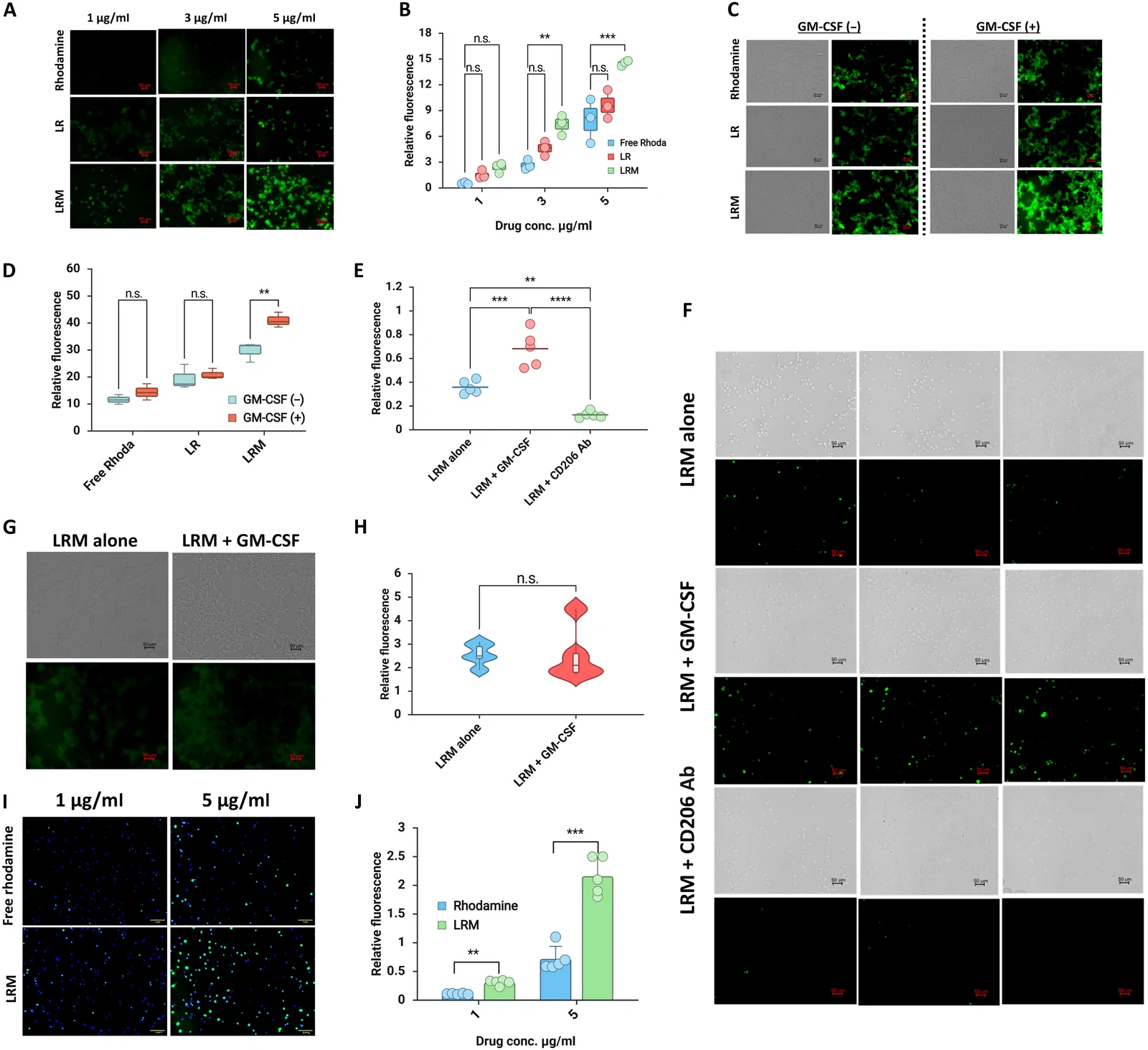
Macrophage and mannose receptor mediated drug uptake. (**A** and **B**) Dose-dependent uptake by RAW264.7 macrophages following 12 hours of incubation at 37°C in Dulbecco’s modified Eagle’s medium (DMEM). Rhodamine (free Rhoda), nonmannitol-derived liposomal rhodamine (LR), and mannitol-engineered liposomal rhodamine (LRM) were compared (scale bars, 50 μm). (**C** and **D**) Drug uptake of RAW264.7 cells stimulated by granulocyte-macrophage colony-stimulating factor (GM-CSF, 0.5 μg/ml, 8 hours) (scale bars, 50 μm). (**E** and **F**) Role of mannose receptor (CD206) in LRM uptake. RAW264.7 cells were pretreated with GM-CSF (50 ng/ml) and subsequently incubated with anti-CD206 antibody (Ab; 0.5 μg/ml) for 6 hours at 37°C before exposure to LRM, assessing receptor-mediated uptake inhibition (scale bars, 50 μm). (**G** and **H**) Macrophage-specific uptake. HEK 293T cells were treated with GM-CSF in the presence of rhodamine formulations (scale bars, 1 μm). (**I** and **J**) Cellular uptake of free rhodamine dye versus mannitol-derived liposomal rhodamine in PMA-differentiated human THP-1 macrophage-like cells. Data are presented as means ± SD (*n* = 3 and 5). Statistical significance: **P < 0.01; ***P < 0.001; ****P < 0.0001; n.s., not significant. See text for detail. This figure was created and resized in high resolution using BioRender. Selaru, F. (2026), https://BioRender.com/h7qtwb0.

To explore the underlying mechanisms driving the enhanced cellular uptake of the mannitol-based liposomal formulation, we stimulated RAW 264.7 cells with granulocyte-macrophage colony-stimulating factor (GM-CSF), a potent activator that promotes macrophage proliferation, differentiation, and functional activation (*20*). We observed a significant increase (*P* < 0.01) in the cellular uptake of the mannitol-derived liposome following 12 hours of incubation at 37°C in the presence of GM-CSF (Fig. 4, C and D). However, no enhancement in uptake was observed for either rhodamine or the nonmannitol-derived liposome (Fig. 4, C and D).

To further confirm the involvement of mannose receptor (CD206)–mediated uptake, we pretreated RAW 264.7 cells with a CD206-specific blocking antibody. Notably, this resulted in a significant reduction (*P* < 0.001) in the cellular uptake of the mannitol-based liposome (Fig. 4E, F, fig. S8), supporting the hypothesis of mannose receptor involvement. In contrast, GM-CSF stimulation did not enhance the uptake of the mannitol-based liposome in human embryonic kidney 293T (HEK 293T) cells (Fig. 4, G and H), further reinforcing the specificity of macrophage targeting. Notably, the mannitol-based liposomal formulation did not exhibit increased uptake in RAW 264.7 cells stimulated with lipopolysaccharide (LPS) (fig. S9, A and B).

We validated the increased cellular uptake using our fluorescently labeled mannitol-derived liposomal formulation in phorbol 12-myristate 13-acetate (PMA)–differentiated THP-1 monocytes, which recapitulate key phenotypic and functional characteristics of human macrophages. After 3 hours of incubation, these experiments demonstrated significantly greater internalization of the liposomal formulation compared with the equivalent free drug concentration (*P* < 0.01, *n* = 3 independent experiments), as quantified by mean fluorescence intensity via fluorescence microscopy (Fig. 4, I and J). These data provide direct evidence supporting mannose receptor–mediated endocytosis as the primary uptake mechanism in human macrophage-like cells, consistent with the ligand density and presentation on our liposomal surface.

Additionally, we observed a significant enhancement in the cellular uptake of the mannitol-based formulation in RAW 264.7 cells pretreated with mannose (fig. S10, A and B), further confirming the role of mannose receptor–mediated drug uptake. Does equivalency in free dye, liposomal rhodamine (LR), and mannitol-based liposomal rhodamine (LRM) formulations group was confirmed by UV-Vis spectroscopy (fig. S11).

### Therapeutic effect of Lipo-tm-HA

To assess the therapeutic efficacy of our liposomal formulation, we established a dextran sodium sulfate (DSS)–induced colitis model, a widely used preclinical model of UC. During the development of the UC model, we rigorously monitored the disease activity index (DAI) and excluded mice that did not exhibit significant colitis symptoms before starting the treatment, as indicated by body weight changes (fig. S12A), hemoccult test results (fig. S12, B and C), stool consistency, and diarrhea. Notably, all mice tested hemoccult-positive after 4 days of receiving 3.5% DSS in drinking water (fig. S12, B and C). The detailed protocol for UC model induction and the treatment regimen is depicted in Fig. 5A.

**Fig. 5.**
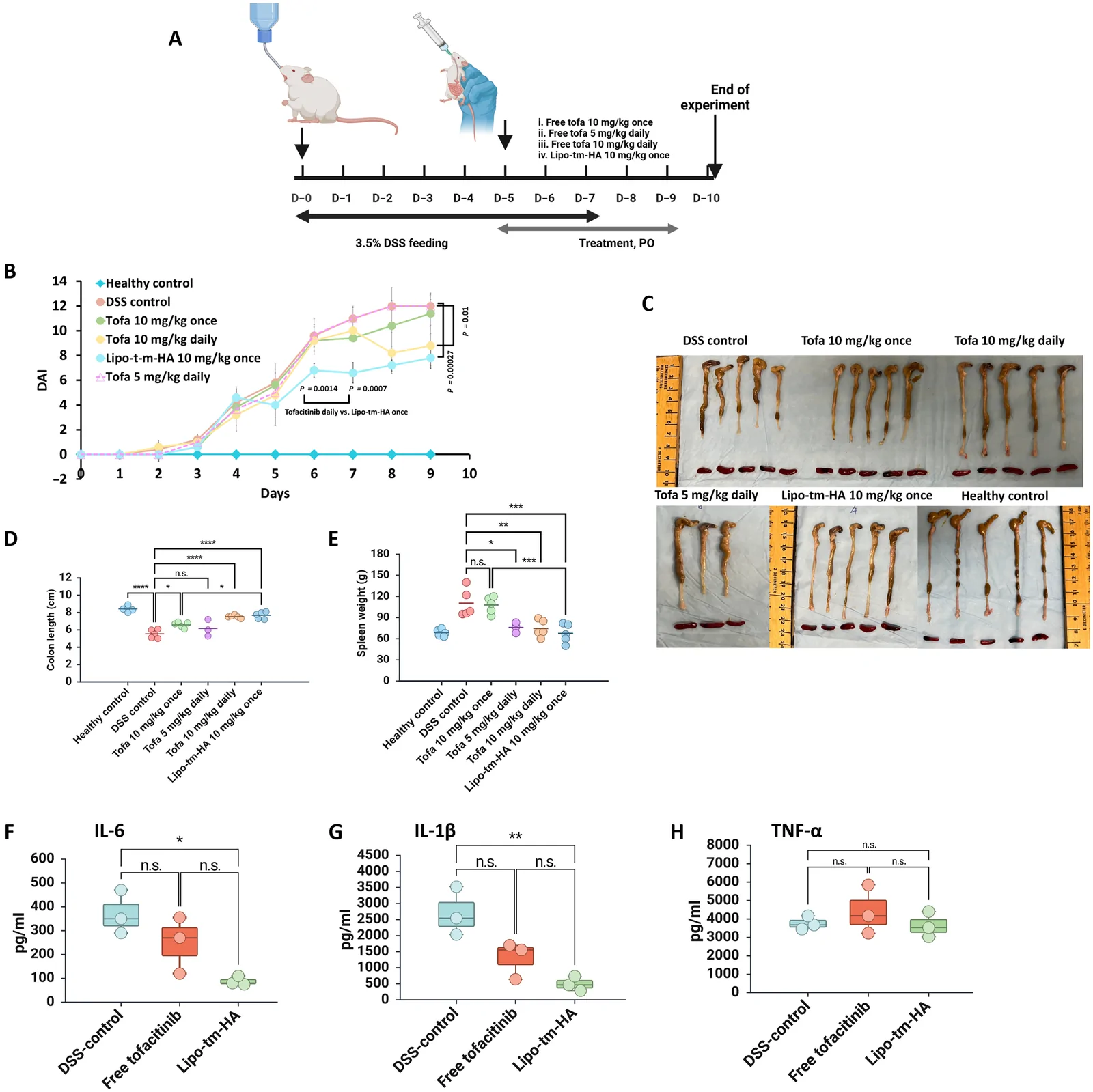
Therapeutic efficacy of Lipo-tm-HA in DSS colitis model. (**A**) Schematic illustration of the experimental procedure for colitis induction and treatment regimen. (**B**) Disease activity index (DAI) progression after treatment with free tofacitinib (Tofa) and Lipo-tm-HA at various doses administered orally (PO), starting from day 5, with the endpoint at day 10. The black bracket under the green line highlights the significant difference between free Tofa (10 mg/kg, daily) and Lipo-tm-HA (10 mg/kg, single dose) on days 6 and 7. (**C**) Representative images of colons and spleens from different treatment groups, including the healthy control (no DSS, no drug treatment). (**D**) Colon length restoration following DSS-induced shortening, with treatment groups showing varying degrees of recovery. (**E**) Splenomegaly assessment showing spleen weight variations across different treatment groups. (**F** to **H**) Cytokine levels in DSS-induced colitis colons following treatment with free tofacitinib and liposomal tofacitinib formulations. (**F**) Interleukin-6 (IL-6), (**G**) IL-1β, and (**H**) tumor necrosis factor–α (TNF-α) concentrations were quantified by enzyme-linked immunosorbent assay (ELISA) from colon homogenates. Lipo-tm-HA significantly reduced IL-6 and IL-1β levels compared with DSS controls. Data are presented as means ± SD (*n* = 3 or 5). Daily tofacitinib, 5 mg/kg; *n* = 3. Statistical significance: *P < 0.05; **P < 0.01; ***P < 0.001; ****P < 0.0001; n.s., not significant. This figure was created and resized in high resolution using BioRender. Selaru, F. (2026), https://BioRender.com/h7qtwb0.

The administration of a single dose of Lipo-tm-HA (10 mg/kg, PO) significantly reduced DAI scores compared with all other groups on days 5 and 6. Notably, a single dose of our Lipo-tm-HA therapeutic demonstrated an equivalent or superior effect on day 9 compared with daily tofacitinib treatment (10 mg/kg, PO) (Fig. 5B). These findings suggest that a single-dose liposomal formulation may be as effective as, or superior to, daily administration of free tofacitinib. Notably, free tofacitinib at 10 mg/kg (single dose) and 5 mg/kg (daily) did not significantly improve DAI scores in the DSS colitis model (Fig. 5B). While the body weight changes following treatment with tofacitinib (10 mg/kg, single dose or daily) as well as with Lipo-tm-HA (10 mg/kg, single dose) did not show a significant improvement compared with the DSS control group (fig. S13), the dose that we have used in the Lipo-tm-HA formulation is ∼30-fold less compared with a previous study that has shown some weight rescue (*21*). Note that blank liposome did not show efficacy on colitis mice (fig. S14).

To further evaluate treatment efficacy, we measured colon length and spleen weight across all treatment groups and the healthy control group on day 10, with representative images shown in Fig. 5C. The untreated DSS control group exhibited significant colon shortening compared with the healthy, noncolitis group, confirming successful colitis induction (Fig. 5, C and D). The treatment with a single dose of Lipo-tm-HA (10 mg/kg) markedly improved colon length compared with the DSS colitis group, as did daily tofacitinib at 10 mg/kg (Fig. 5, C and D). In contrast, a single dose of tofacitinib at 10 mg/kg or a daily dose of tofacitinib at 5 mg/kg failed to restore colon length, a daily dose of tofacitinib at 10 mg/kg improved colon length (Fig. 5, C and D). Splenomegaly, a hallmark of colitis (*22*), was observed in mice with DSS-induced colitis, indicating colonic inflammation. Following treatment with our liposomal formulation, spleen weight was significantly reduced compared with that in the group of mice with DSS-induced colitis, with an effect comparable to daily tofacitinib treatment which sees the administration of tofacitinib at a dose fivefold larger (Fig. 5, C and E).

We performed additional pharmacodynamic evaluations in the colitis model. Specifically, we quantified key proinflammatory cytokines [interleukin-6 (IL-6), IL-1β, and tumor necrosis factor–α (TNF-α)] in colonic tissue and compared the effects of free tofacitinib with our Lipo-tm-HA formulation. These data demonstrate that a single oral dose of Lipo-tm-HA (10 mg/kg) produced a more pronounced suppression of colonic inflammatory cytokines such as IL-6 and IL-1β than daily oral dosing of free tofacitinib at the same nominal dose (Fig. 5, F and G). We also observed that both free tofacitinib and Lipo-tm-HA did not show any effect on TNF-α production (Fig. 5H). This selective suppression of IL-6 and IL-1β without affecting TNF-α likely reflects differential signaling pathway regulation as IL-6 and IL-1β expression can be modulated independently of TNF-α through distinct transcription factor activation, such as signal transducer and activator of transcription 3 and nuclear factor κB pathways (*23*, *24*). These findings further support that Lipo-tm-HA preferentially delivers tofacitinib to the inflamed colon and enhances local anti-inflammatory activity compare to the free drug.

Last, we assessed the effect on mucosal injury using hematoxylin and eosin (H&E) staining, with representative histologic images shown in Fig. 6A. DSS treatment alone resulted in extensive mucosal injury, which included ulceration, severe leukocyte infiltration into the lamina propria, and epithelial hyperplasia. Treatment with our liposomal formulation led to significant (*P* < 0.05) reduction in mucosal injury and was the only treatment arm that led to a statistically significant decrease in mucosal injury compared with the untreated group with DSS-induced colitis (Fig. 6B). The daily tofacitinib treatment trended in the direction of reduced mucosal injury; however, the effect was not statistically significant (Fig. 6B). Collectively, these findings indicate that our liposomal formulation offers improved colitis treatment at a significant fivefold reduced dose when compared with daily dosing of tofacitinib (Fig. 6B). We also measured the CD45^+^ total leukocyte as well as alteration of M1/M2 macrophage in the inflamed colon. Our data suggest that the Lipo-tm-HA significantly (*P* < 0.001) reduced the total leukocyte however did not alter the M1/M2 population by flow cytometry (Fig. 6C).

**Fig. 6.**
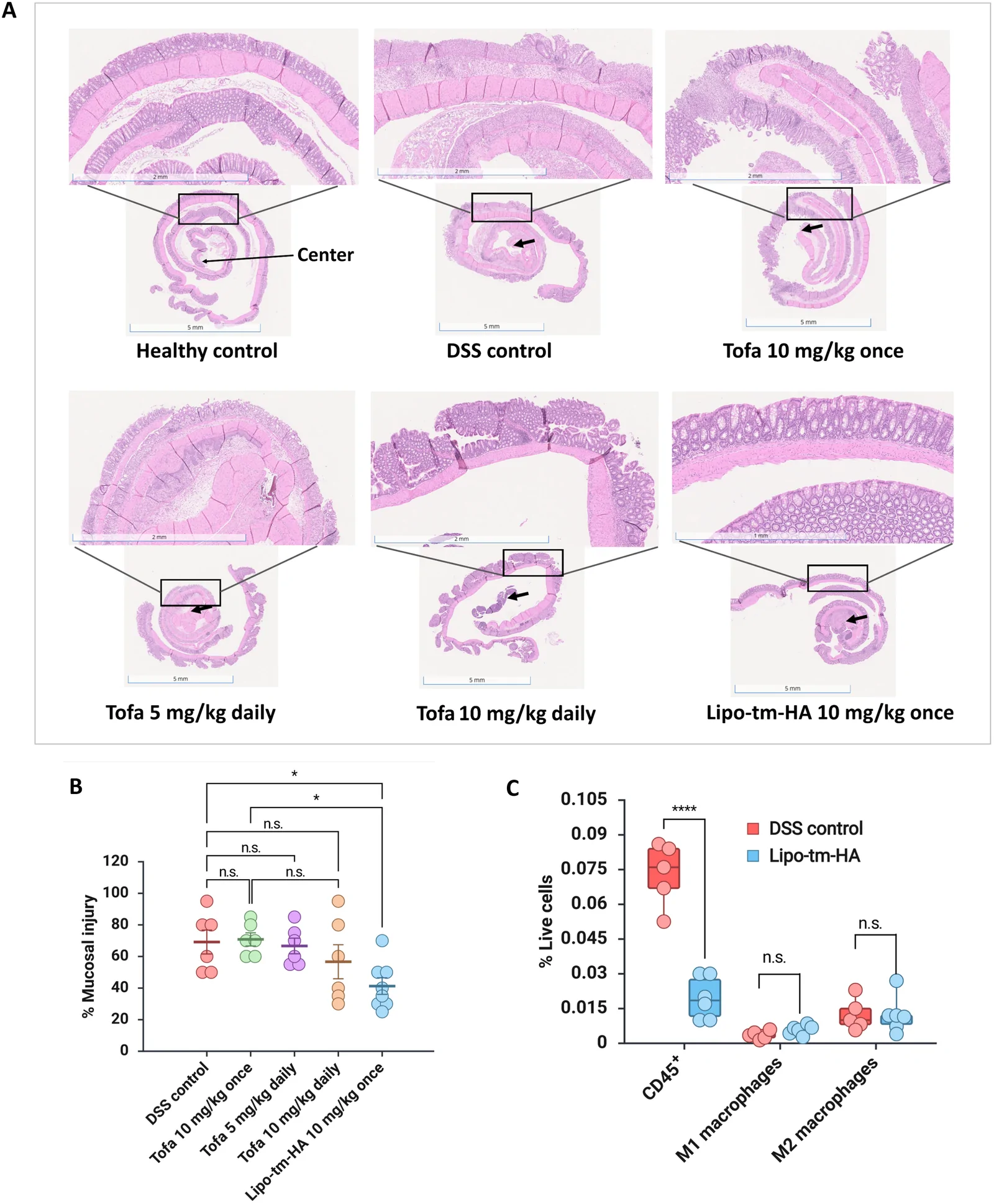
Therapeutic efficacy of Lipo-tm-HA in DSS-induced colitis assessed by H&E histology and flow cytometry. (**A**) Representative hematoxylin and eosin (H&E)–stained Swiss roll sections of colons from different treatment groups, with expanded high-magnification views of selected regions. The anorectum is at the center of the Swiss roll indicated by black arrows. The DSS colitis model was established as detailed in Fig. 5A, followed by therapeutic intervention. H&E staining was performed following standard protocols at the Johns Hopkins University core facility. (**B**) Quantitative analysis of mucosal injury scores across treatment groups. Only Lipo-tm-HA treatment resulted in a statistically significant reduction in mucosal damage compared with DSS controls (*P < 0.05). (**C**) Flow cytometric analysis of immune cell infiltration in DSS colitis colons posttreatment. Lipo-tm-HA therapy significantly reduced CD45^+^ leukocyte infiltration (****P < 0.0001), while M1/M2 macrophage populations remained unaltered (n.s.). Data are presented as means ± SD (*n* = 6 to 8 mice per group). Statistical significance: *P < 0.05; ****P < 0.0001; n.s., not significant*.* This figure was created and resized in high resolution using BioRender. Selaru, F. (2026), https://BioRender.com/h7qtwb0.

### Biodistribution, systemic exposure, and toxicity study

We hypothesized that our liposomal formulation would selectively target colonic inflammation while minimizing systemic exposure. To validate this concept, we conducted a comparative tissue distribution study between rhodamine-labeled liposomal formulation (Lipo-rm-HA) and free rhodamine dye in the DSS colitis model, using ex vivo imaging systems. Confirming both our hypothesis, our findings revealed that Lipo-rm-HA preferentially accumulated in the colon 24 hours post–oral administration, with minimal distribution to other organs, including the heart, lungs, liver, spleen, and, kidneys (Fig. 7, A and B). Representative tissue images and ex vivo fluorescence imaging results are shown in fig. S15A and Fig. 7A. Notably, colon-specific accumulation of Lipo-rm-HA was ∼5- to 8-fold higher than that of free rhodamine dye at 24 hours (Fig. 7B). Furthermore, Lipo-rm-HA exhibited negligible or undetectable accumulation in noncolonic tissues (Fig. 7B). Encouragingly, Lipo-rm-HA accumulation in healthy, noncolitis mice was significantly lower or undetectable after 24 hours, as confirmed by ex vivo imaging (fig. S15B). Moreover, no detectable fluorescence signal was observed in blood or stool samples across all groups after 24 hours, regardless of colitis status or formulation type (fig. S15, C and D).

**Fig. 7.**
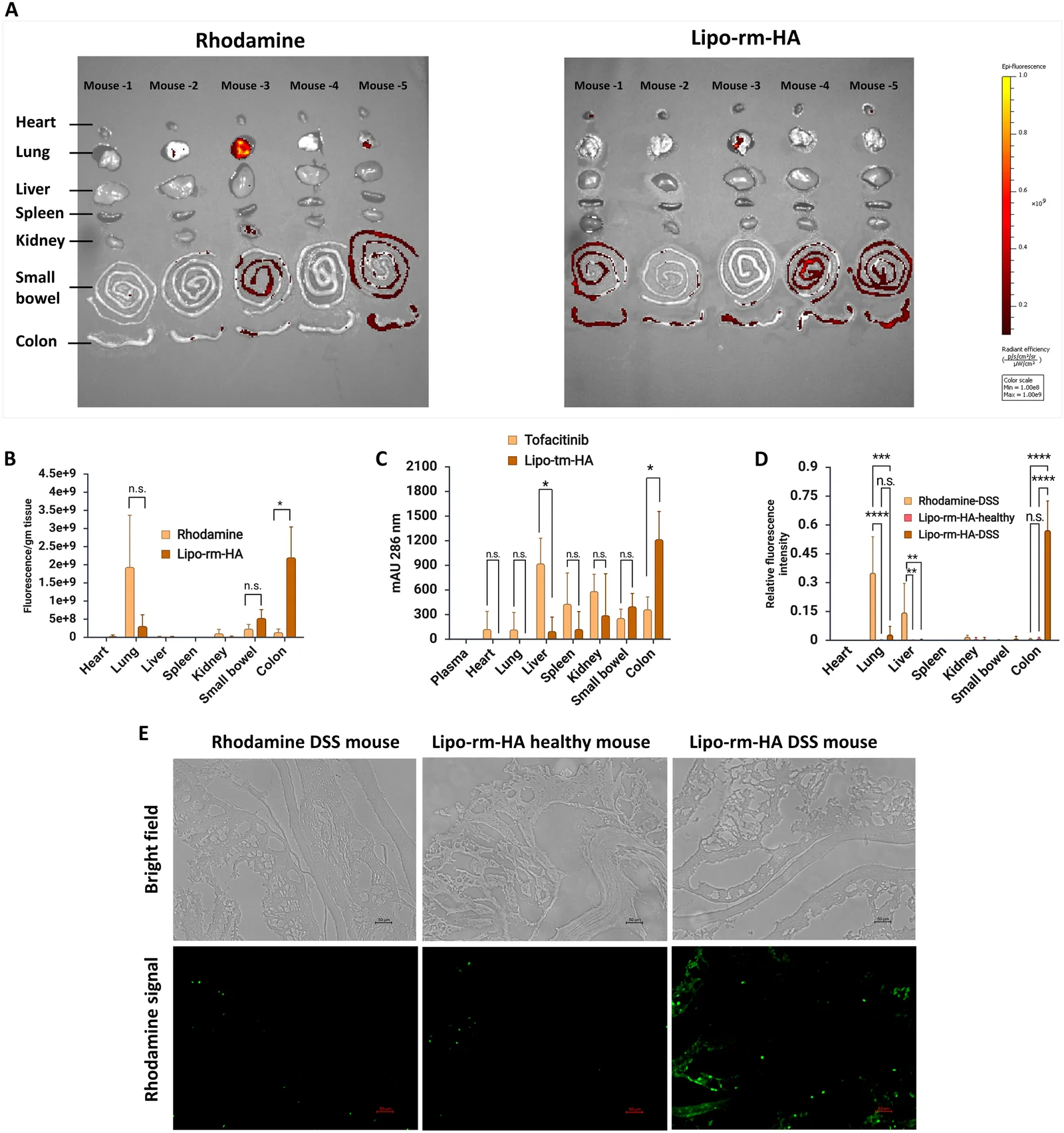
Colon inflammation-selective accumulation of Liposomal formulation. (**A**) Ex vivo fluorescence imaging comparing the biodistribution of free dye rhodamine (left) and mannitol-engineered liposomal rhodamine coated with hyaluronic acid (Lipo-rm-HA) (right) in DSS-induced colitis mice. Rhodamine and Lipo-rm-HA were administered orally (PO) at a dose of 2.5 mg/kg (rhodamine equivalent). (**B**) Quantitative analysis of fluorescence signals in different tissues, comparing free rhodamine and Lipo-rm-HA. (**C**) Comparison of biodistribution of free tofacitinib and Lipo-tm-HA in DSS colitis mice measured by reverse phase chromatography in C-18 column. (**D**) Quantification of colon tissue localization of free dye versus liposomal formulation. (**E**) Fluorescence microscopy images of colon tissue sections in colitis mice treated with free dye and Lipo-rm-HA. Colon tissues were fixed with 10% formalin, embedded in O.C.T. blocks, cryo-section (10 μm thick). Green fluorescence indicates rhodamine signal (scale bars, 50 μm). Data are presented as means ± SE (*n* = 5). Statistical significance: *P < 0.05; **P < 0.01; ***P < 0.001; ****P < 0.0001; n.s., not significant. This figure was created and resized in high resolution using BioRender. Selaru, F. (2026), https://BioRender.com/h7qtwb0.

We further quantified the direct tissue concentration of tofacitinib, in addition to the fluorescence signal, in the colon and other major organs. For this purpose, we developed and validated an HPLC method to directly measure tofacitinib in tissues collected from DSS-colitis mice. Our results showed that the tofacitinib-loaded Lipo-tm-HA formulation preferentially accumulated in the inflamed colon following oral administration, whereas free tofacitinib exhibited relatively greater distribution to systemic organs, including the liver, spleen, and kidney (Fig. 7C). These findings demonstrate that the liposomal formulation enhances local drug delivery to the diseased colon while limiting off-target systemic exposure.

To further investigate tissue localization, we performed formalin fixation, cryo-sectioning, and fluorescence microscopy. Consistent with our ex vivo imaging results, strong fluorescence signals were exclusively observed in colonic tissue, with negligible fluorescence in other organs (Fig. 7, D and E, and fig. S16). In contrast, free rhodamine dye primarily accumulated in the lungs and liver (Fig. 7D and fig. S16). These findings demonstrate that our liposomal formulation selectively targets inflamed colonic tissue, highlighting its potential for localized drug delivery in colitis treatment while minimizing off-target distribution.

We next evaluated the systemic exposure of our fluorescently labeled liposomal formulation and observed that the free low–molecular-weight dye rapidly entered the bloodstream following oral administration, with a notable fluorescence signal detected in the blood within 30 min, indicating immediate systemic absorption. In contrast, the Lipo-rm-HA formulation exhibited a markedly reduced plasma exposure, ∼4- to 5-fold lower than that of the free rhodamine after 24 hours of drug administration (Fig. 8, A and B). No significant differences in stool drug concentrations were observed 24 hours after administration (Fig. 8C).

**Fig. 8.**
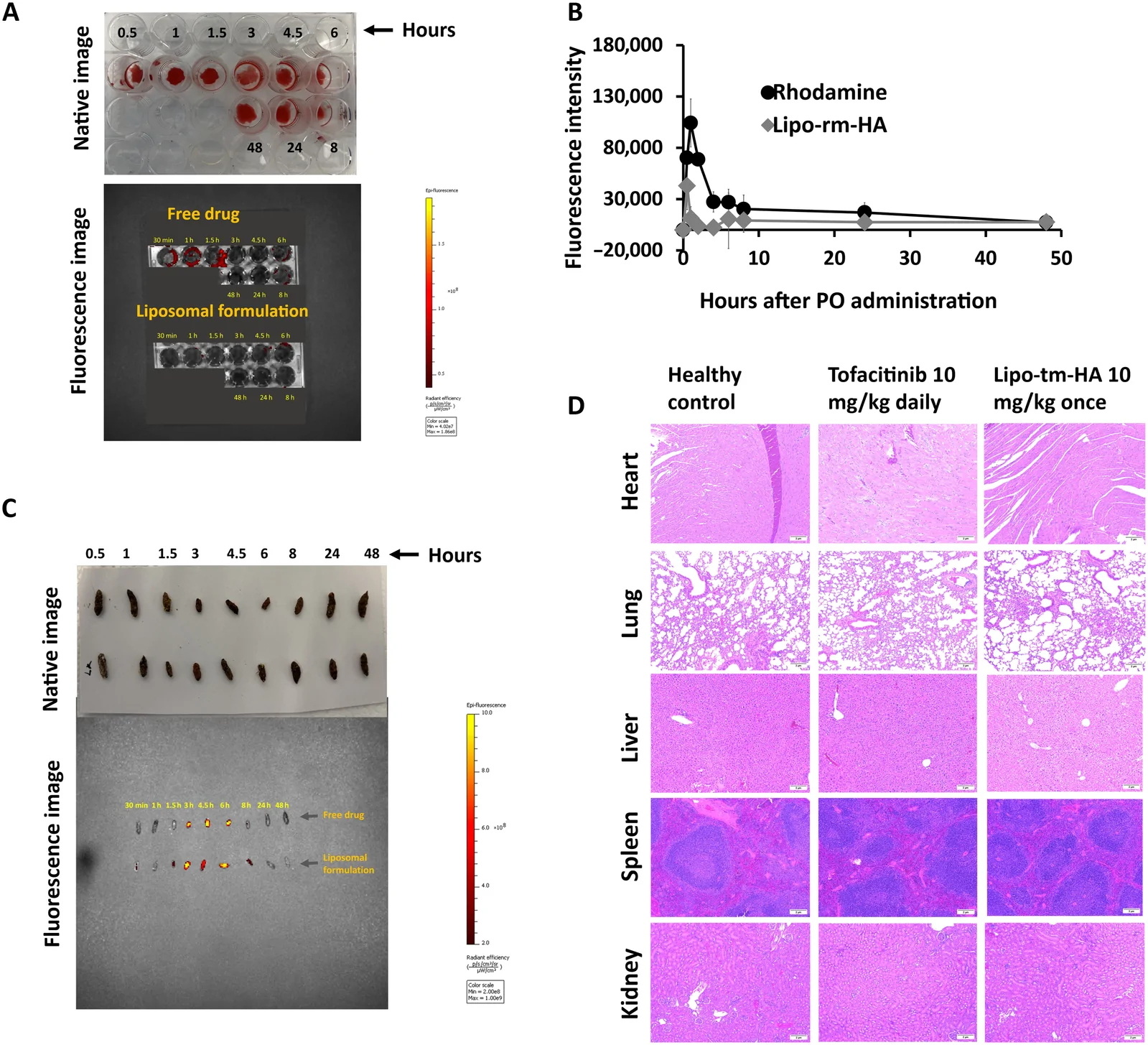
Systemic exposure and toxicity study of Lipo-tm-HA. (**A**) Representative in vivo fluorescence imaging (IVIS) showing systemic exposure of free dye rhodamine and mannitol-engineered liposomal rhodamine coated with hyaluronic acid (Lipo-rm-HA) in C57BL/6 mice after oral administration (PO). (**B**) Comparison of fluorescence signal after PO administration of rhodamine and Lipo-rm-HA in blood, indicating significantly lower systemic exposure of Lipo-rm-HA compared with free dye. (**C**) Time-dependent fluorescence signal in stool samples of Lipo-rm-HA versus free rhodamine examined by IVIS. h, hours. (**D**) Toxicity evaluation of free tofacitinib and Lipo-rm-HA in major organs (heart, lung, liver, spleen, and kidney) by H&E staining following oral administration at dose 10 mg/kg, PO (scale bars, 2 μm). Data are presented as means ± SD (*n* = 5). This figure was resized in high resolution using BioRender. Selaru, F. (2026), https://BioRender.com/h7qtwb0.

We further tested whether Lipo-tm-HA has additional toxicity when compared with tofacitinib in a cell intrinsic fashion. We found that Lipo-tm-HA displayed the same level of toxicity as tofacitinib in two cell lines tested, arguing that the additional ingredients in the Lipo-tm-HA does not increase toxicity (fig. S17, A and B). We then tested whether there was any evidence of end organ injury by performing H&E staining histopathologic experiment of various tissues. We found no significant toxicity in major organs such as the heart, liver, spleen, and kidney in both the free tofacitinib and Lipo-tm-HA treatment groups (Fig. 8D). Notably, one of the mice in the free tofacitinib group showed a small focus of acute pneumonia, foci of chronic inflammation in the lung parenchyma, and the presence of macrophages within the parenchyma engaged in clearing fresh hemorrhage (Fig. 8D).

## DISCUSSION

The clinical management of IBD remains challenging due to multiple factors, including heterogeneity of disease phenotypes, patient response to therapeutics, inability to precisely predict which patient population responds to any individual drug, as well as limiting side effects and potential complications (*3*). Tofacitinib, a JAK small-molecule inhibitor, represents a promising oral alternative to biologic therapies for IBD, demonstrating efficacy in modulating immune signaling pathways, particularly in the management of UC (*5*). However, its use is limited by substantial safety concerns, associated with a black box warning, including an increased risk of infections, thromboembolism, and potential malignancies, necessitating careful patient selection and monitoring (*11*, *25*). In addition, tofacitinib is avoided in pregnancy and used cautiously in women of childbearing age due to possible teratogenic effects, severely restricting treatment access for this patient population (*25*). The rapid onset of action of tofacitinib (when compared with some of the biologic agent alternatives) and its effectiveness including in cases where the inflammation is severe, such as cases of Acute Severe Ulcerative Colitis, represent great opportunities that cannot be used fully in young women. There is a great clinical need to increase addressability of tofacitinib for patients with IBD by improving its safety profile.

Here, we hypothesized that a targeted delivery of tofacitinib will increase the local colonic bioavailability of the drug at the site of inflammation. By extension, this approach will increase the effectiveness of tofacitinib while simultaneously decreasing systemic exposure, which is responsible for its side effects. To test this hypothesis, we leveraged formulation and materials engineering expertise to increase the delivery of tofacitinib to areas of inflammation and specifically to macrophages within those areas. This approach will allow reducing the total dose of tofacitinib given as well as the systemic exposure. In selecting a delivery platform to meet our delivery requirements, we focused on methodologies known to be safe in clinical-grade applications, with the goal of increasing the translatability of our findings.

The physicochemical properties of Lipo-tm-HA, including purity, size, PDI, and surface charge, are presented in Fig. 1 and fig. S1. Notably, particle size plays a critical role in behavior as drug carriers. For example, nanoparticles sized 50 to 200 nm are optimal for systemic circulation and enhanced mucosal penetration as they effectively traverse biological barriers while evading rapid clearance by macrophages (*26*). In contrast, particles within the 200- to 500-nm range are ideal for colon-targeted drug delivery (*27*) as their larger size promotes prolonged retention in inflamed tissues and preferential uptake by activated immune cells. However, particles exceeding 500 nm face limited diffusion through the intestinal mucus layer, reducing their ability to effectively reach inflamed sites (*26*, *27*). Additionally, immune cells such as macrophages and dendritic cells preferentially internalize sub-500-nm particles, rendering 1000-nm formulations suboptimal for intracellular drug delivery (*28*). Furthermore, larger particles are more susceptible to peristaltic clearance, diminishing their retention at inflamed sites and ultimately reducing therapeutic efficacy (*28*). Taking these considerations into account, we designed our liposomal formulation Lipo-tm-HA to exhibit a size range of 280 to 300 nm, making it well suited for UC treatment.

Stability and controlled drug release are critical determinants of a successful drug delivery system. A significant number of nanomedicines have failed in clinical trials due to insufficiently calibrated stability and/or inadequate release of active pharmaceutical ingredient (API) (*29*). For instance, following intravenous or oral administration, APIs that are either covalently conjugated to polymeric carriers or encapsulated within nanocarriers may prematurely dissociate in biological fluids, such as blood or the gastric environment (*29*). This instability causes nanomedicines to behave similarly to low–molecular-weight drugs, thereby negating the intended benefits of nanotechnology-based formulations (*29*). In contrast, certain nanomedicines, such as liposomal doxorubicin formulations (Doxil), exhibit excessive stability, which hinders effective drug release at the tumor site, ultimately leading to suboptimal therapeutic efficacy (*30*).

Among various drug administration routes, the oral delivery is preferred because of ease of administration and patient compliance (*31*). However, many nanoformulations and oral drugs have failed in the FDA approval process due to instability and inadequate drug release, which compromise efficacy and safety (*32*), a major ongoing challenge in drug development and regulatory science (*32*, *33*). For instance, oxycodone (OxyContin) and other oral opioids have faced FDA scrutiny and, in some cases, market withdrawal or restricted approval due to dose dumping, a form of drug release failure in which rapid release under specific conditions can lead to acute toxicity (*34*). Our liposomal formulation, Lipo-tm-HA, exhibits enhanced structural stability in the gastric environment, attributed to the protective HA coating (Fig. 2 and figs. S3 and S4). Additionally, it enables controlled and site-specific drug release within the intestinal milieu, optimizing therapeutic delivery (Fig. 2). HA coating was selected for its biocompatible and biodegradable properties, ensuring safe and targeted drug delivery with minimal toxicity and immunogenicity (*35*). Moreover, its mucoadhesive properties enhance drug retention at the site of action while maintaining excellent biostability, making it a safe and effective excipient in advanced pharmaceutical formulations (*36*).

Our formulation was strategically designed to target transferrin within the inflamed colonic mucosa for enhanced drug delivery to the site where it is needed. Inflammation causes antigenic stimulation which in turn triggers increased capillary permeability, leading to the extravasation of transferrin into the inflamed colonic tissue (*37*). Subsequently, transferrin leaks into the gut lumen, where it undergoes protonation, acquiring a positive charge, which may facilitate its interaction with negatively charged molecule and enhance targeted drug localization (*37*). Our liposomal formulation was specifically designed to exhibit a negative charge (Fig. 1D). Validating our hypothesis, coincubation of the liposomal formulation with transferrin resulted in charge neutralization, and binding was further confirmed using fluorescently labeled transferrin through ultracentrifugation (Fig. 3). These data are consistent with the previous report that negatively charged liposomes with transferrin profoundly attenuated their adherence to the inflamed mucosa of the rat colon (*37*). This finding suggests that electrostatic interactions and transferrin-mediated targeting play a critical role in promoting site-specific retention of the liposomal formulation in inflammatory conditions.

The liposomal formulation that we report here was optimized for careful targeting of inflamed areas. To achieve this goal, we implemented a dual targeting functionality aims at colonic inflammation. In addition to targeting positively charged areas of the bowel, we aimed to target macrophages and increase the specificity of tofacitinib delivery while facilitating macrophage uptake, thereby optimizing therapeutic efficacy in the inflammatory microenvironment. Macrophages play a pivotal role in inflammation, acting as key immune regulators by orchestrating proinflammatory (M1) and anti-inflammatory (M2) responses in tissue homeostasis and disease progression (*38*). During inflammation, activated macrophages secrete cytokines, chemokines, and reactive oxygen species, driving either immune activation and tissue damage (M1 phenotype) or resolution and repair (M2 phenotype), depending on the microenvironmental cues (*38*). Our liposomal formulation showed macrophage mediated uptake through mannose receptor evaluated by stimulating macrophage cells with GM-CSF and blocking the mannose receptor by CD206 antibody (Fig. 4 and figs. S8 to S11). We hypothesize that the enhanced macrophage-mediated uptake observed with our liposomal formulation is attributed to the mannitol modification, as this effect was absent in both the free drug and nonmannitol-based liposomal formulations. Furthermore, the structural similarity between mannitol and mannose may facilitate mannose receptor–mediated uptake.

Notably, while LPS activation did not increase drug uptake in RAW 264.7 macrophages, GM-CSF significantly enhanced the cellular uptake of the mannitol-based liposomes (Fig. 4 and fig. S9). This difference likely stems from the distinct effects of GM-CSF and LPS on macrophage function: GM-CSF enhances endocytosis and phagocytosis by promoting macrophage differentiation, up-regulating scavenger receptors, and increasing membrane trafficking, thereby facilitating greater drug internalization (*20*). In contrast, LPS, as a Toll-like receptor 4 agonist, induces a proinflammatory state, redirecting cellular resources toward cytokine secretion and immune signaling rather than receptor-mediated endocytosis (*39*).

Mannose pretreatment increased the uptake of our mannitol-modified liposomes in RAW 264.7 cells (fig. S10), likely due to mannose binding to the mannose receptor on the macrophage surface, thus enhancing the internalization of mannose-modified drug carriers, a strategy commonly used in macrophage-targeted drug delivery (*40*). Prior work found that pretreatment with mannose up-regulates mannose receptor expression either through receptor recycling (*41*, *42*) or through compensatory endocytosis pathways (*43*). We aimed to investigate whether we can modulate uptake of our nanoparticle thorough this mechanism. To this end, we pretreated cells with mannose and measured uptake of the nanoparticle. While mannitol is conventionally used as a stabilizing excipient in pharmaceutical formulations, its specific interaction with liposomes and the underlying mechanism by which it enhances liposomal uptake in RAW 264.7 cells remain incompletely understood. Further investigation is required to elucidate whether mannitol-mediated structural modifications or ligand-receptor interactions contribute to the improved cellular internalization and targeting efficiency of the liposomal formulation.

We evaluated our liposomal formulation (Lipo-tm-HA) in the DSS-induced colitis model, a well-established preclinical model that replicates major pathological features of UC, including epithelial barrier disruption, immune cell infiltration, and heightened inflammatory cytokine release (*44*). The DSS model’s reproducibility and dose-dependent disease induction enable precise assessment of drug efficacy, colon-targeted delivery, and mucosal healing under controlled conditions (*44*). Our findings demonstrate that a single oral dose (10 mg/kg) of Lipo-tm-HA is comparable or superior to daily treatment free tofacitinib, as evidenced by body weight preservation, DAI score reduction, colon length restoration, decreased splenomegaly, inflammatory cytokines, and lower colonic mucosal injury (Figs. 5, and 6). Notably, treatment with Lipo-tm-HA displayed marked protective effects against inflammation induced colon shortening in the DSS model (Fig. 5, C and D), indicating effective mucosal protection and tissue repair. Furthermore, splenomegaly, a hallmark of colitis-associated systemic immune activation (*45*), was significantly attenuated in the Lipo-tm-HA-treated group, suggesting a reduction in systemic inflammation (Fig. 5, C and E). Collectively, these results highlight the selective targeting capability of our liposomal formulation, which results in higher doses at the site of inflammation while protecting the systemic circulation. In contrast, free tofacitinib exhibited broader systemic distribution with reduced localized efficacy.

We evaluated the colon-localized delivery of our liposomal formulation using both rhodamine as a tool drug and direct tofacitinib accumulation in the colon. Both ex vivo imaging and fluorescence microscopy, as well as tofacitinib biodistribution analysis, showed that Lipo-tm-HA accumulated preferentially in the inflamed colon at levels ∼5- to 8-fold higher than free rhodamine dye in the DSS colitis model (Fig. 7). In contrast, healthy mice showed no significant drug accumulation in the colon (fig. S16), further confirming the inflammation-targeted delivery of the liposomal formulation. These data correlate well with the observed dose-specific therapeutic efficacy, wherein a single oral dose of Lipo-tm-HA (10 mg/kg) demonstrated superior or comparable outcomes to daily administration of free tofacitinib given at a dose of 10 mg/kg daily for 5 days. This correlation provides with a mechanistic explanation of equal or higher efficacy of the liposomal formulation which is administered at fivefold lower overall dose than free drug but accumulates in the colon at a five- to eightfold higher rate.

Pharmacokinetic study with rhodamine-labeled liposomes revealed significantly lower systemic exposure compared with free rhodamine dye (Fig. 8, A and B), highlighting the formulation’s enhanced selectivity and reduced off-target distribution. Of interest to future clinical translation, the rodent group treated with free tofacitinib exhibited histopathological signs of pulmonary toxicity in two animals, including small foci of acute pneumonia, chronic inflammatory infiltrates in the lung parenchyma, and the presence of macrophages engaged in the clearance of recent hemorrhagic events (Fig. 8D). These findings may be consistent with previous reports indicating that tofacitinib is associated with an increased risk of pulmonary adverse effects, including pulmonary embolism, respiratory tract infections such as pneumonia, and interstitial lung disease (*12*, *46*, *47*). In sharp contrast, in addition to increased efficacy of the Lipo-tm-HA, we observed no lung complications as in the control group. To establish the safety profile of our liposomal formulation compared with free tofacitinib, further studies are needed.

Collectively, the data reported here suggest that Lipo-tm-HA could potentially reduce dosing frequency, unlike the daily administration of free tofacitinib, offering a more convenient and sustained therapeutic option. Furthermore, by minimizing off-target effects and systemic exposure, the liposomal formulation may mitigate the adverse effects associated with tofacitinib, which carries an FDA black box warning. Taken together, these findings demonstrate the feasibility of an inflammation-targeting platform for tofacitinib (or any JAK inhibitor), addressing a critical unmet clinical need in IBD therapy.

## MATERIALS AND METHODS

### Study design

This study aimed to develop a colon-targeted drug delivery system for IBD, with a particular focus on UC, by engineering a liposomal platform modified with mannitol and coated with HA. Mannitol was incorporated to facilitate active targeting of macrophages via the mannose receptor (CD206), whereas the HA coating was designed to enhance oral delivery by protecting the formulation from degradation in the gastric environment. The platform was used to encapsulate tofacitinib, a small-molecule JAK inhibitor with known efficacy that is unfortunately limited in some cases (particularly young women of child bearing age) due to safety concerns. To thoroughly evaluate the formulation, we conducted comprehensive physicochemical characterizations, including assessments of drug purity, particle size, drug loading efficiency, colloidal stability, and in vitro drug release kinetics. All analytical assays were performed in triplicate using advanced analytical techniques. Drug release and stability were assessed in simulated gastric and intestinal fluids to mimic the physiological environment of the gastrointestinal tract. To explore transferrin-liposome interactions, both fluorescently labeled and unlabeled transferrin were used. Targeted uptake via the mannose receptor was evaluated in RAW 264.7 macrophages, which were activated using GM-CSF, LPS, and mannose, and receptor-specific uptake was confirmed via CD206 antibody blockade.

Therapeutic efficacy was assessed in vivo using a DSS-induced colitis model in C57BL/6 mice (3.5% DSS). Disease progression and treatment response were evaluated via body weight monitoring, DAI, colon length measurement, splenomegaly, inflammatory cytokines, and histological quantification of ulceration. Biodistribution studies in healthy and colitis-induced mice were conducted using fluorescently labeled liposomes to validate inflammation-targeted drug accumulation. These results were corroborated using in vivo imaging system (IVIS) and fluorescence microscopy. Pharmacokinetics and systemic exposure were investigated by comparing the circulation and fecal excretion profiles of the fluorescently labeled liposomal formulation as well as tofacitinib-liposomal formulation. Cytotoxicity of the formulation was evaluated in HEK 293T, THP-1, and RAW 264.7 cells, and systemic toxicity was assessed via histological analysis of major organs following in vivo administration. For all in vivo studies, animals were randomly assigned to treatment and control groups. Sample sizes were determined through power analysis based on preliminary efficacy studies, ensuring a minimum of five animals per group. Mice were monitored daily and humanely euthanized at designated endpoints. All animal experiments were conducted in accordance with the guidelines of the Institutional Animal Care and Use Committee of Johns Hopkins University, under approved protocol number MO22M319.

### Preparation of liposomal formulation

The tofacitinib-encapsulated liposomal formulation was prepared using the conventional thin-film hydration method. Briefly, 7 mmol of distearoylphosphatidylcholine, 3 mmol of cholesterol, and 20% (w/w) of tofacitinib were dissolved in chloroform and mixed in a conical flask equipped with a magnetic stirrer. The mixture was incubated at 60°C for 15 to 20 min to ensure complete solubilization, followed by solvent removal under reduced pressure using a rotary evaporator at 40°C. The resulting lipid film was further dried overnight in a vacuum oven at 60°C to remove any residual solvent. Subsequently, the dried lipid film was hydrated with 5 ml of deionized water containing 15 to 20% (w/w) d-mannitol. The hydration process was carried out at 60°C for 30 min, followed by brief vortexing (∼3 min) in a 50-ml Falcon tube to promote dispersion. The liposomal suspension was then extruded through a 200-nm polycarbonate membrane using an Avanti extruder maintained at 60°C. The extrusion was repeated five to six times by passing the suspension between syringes to ensure the formation of a uniform and homogeneous liposomal formulation. The liposomal dispersion was subsequently dialyzed using Visking dialysis tubing (MW, ∼10 kDa) against deionized water to remove unencapsulated drug and excess reagents and then lyophilized the liposomal formulation with mannitol and tofacitinib (Lipo-tm) for storage.

For HA coating, a one-pot synthesis method was used. Approximately 100 mg of the lyophilized liposomal powder was dissolved in 50 ml of deionized water, followed by the addition of 8 mg of hyaluronic acid. The mixture was stirred magnetically for 5 min before the addition of 2 mg of sodium borohydride to facilitate reductive conjugation. The reaction mixture was stirred at room temperature for 24 hours. Postreaction, the HA-coated liposomes were dialyzed against deionized water using Visking tubing and lyophilized the HA coated liposomal formulation (Lipo-tm-HA) for further use.

### Characterization of Lipo-tm-HA

#### Dynamic light scattering

Different liposomal formulations Lipo-t, Lipo-tm, and Lipo-tm-HA were diluted in deionized water to a final concentration of 2 mg/ml and filtered through a 0.2-μm syringe filter before analysis. DLS measurements were performed at 25°C using an electrophoretic light-scattering analyzer, TTEC Zetasizer Pro (Malvern Panalytical, Malvern, UK). Particle size and PDI were determined using both the cumulant analysis and intensity-weighted histogram methods. Zeta potential measurements were conducted to evaluate the surface charge of the formulations before and after HA coating. Samples were prepared at a concentration of 5 mg/ml in deionized water, and surface charged was measured using the same instrument.

#### Transmission electron microscopy

Lipo-t, Lipo-tm, and Lipo-tm-HA were dissolved in deionized water at a concentration of 5 mg/ml. Sample solution (50 μl) was mixed with 50 μl of 0.01% (w/v) phosphotungstic acid on a piece of parafilm. The resulting mixture was carefully transferred onto a carbon-coated copper grid (Millipore Sigma-Aldrich, MO, USA). The grid was then vacuum dried overnight at room temperature. TEM imaging was performed using a FEI Talos F200C FEG transmission electron microscope (Thermo Fisher Scientific, USA) operated at an accelerating voltage of ∼80 kV to assess the shape and size.

### Stability of Lipo-tm-HA in gastric and intestinal fluid

Lipo-tm and Lipo-tm-HA were dissolved at a concentration of 2 mg/ml in simulated gastric fluid, simulated intestinal fluid, and deionized water. The samples were incubated at 37°C to mimic physiological conditions. The hydrodynamic diameter and PDI of the liposomal formulations were measured at predetermined time points—10 min, 20 min, 40 min, 1 hour, 6 hours, 24 hours, 48 hours, and 120 hours—using DLS, as previously described.

### In vitro tofacitinib release from Lipo-tm-HA

Lipo-tm-HA was dissolved at a concentration of 5 mg/ml in gastric and intestinal fluid and deionized water. Approximately 5 ml of each formulation was transferred into individual Visking dialysis tubes (molecular weight cutoff, ∼10 kDa), which were then placed into 50-ml Falcon tubes containing 30 ml of the corresponding external medium (gastric fluid, intestinal fluid, and deionized water). The tubes were incubated at 37°C under continuous shaking conditions. In this setup, intact Lipo-tm-HA remained inside the dialysis membrane, whereas free (released) tofacitinib was able to diffuse into the external medium (see Fig. 2E). At predetermined time points, aliquots of the external solution were collected, and the amount of released tofacitinib was quantified by UV-Vis spectroscopy. The percentage of drug release was calculated on the basis of the initial concentration of tofacitinib encapsulated within the liposomal formulation inside the dialysis tube.

### Binding of Lipo-tm-HA to transferrin

Lipo-tm-HA was mixed with transferrin at weight ratios of 1:0.1, 1:0.5, and 1:1 in an acidic buffer (pH 5.0) and incubated at 37°C for 30 min to facilitate surface interaction. Following incubation, each mixture was centrifuged at 12,000*g* for 15 min to pellet the liposomes, while unbound transferrin remained in the supernatant. The supernatant was discarded, and the resulting pellet was redispersed in deionized water. To ensure thorough removal of unbound transferrin, the washing step was repeated five times using ultracentrifugation under the same conditions. The final pellet was reconstituted in deionized water, and the surface charge (zeta potential) and particle size of the liposomal formulations were measured using DLS.

### Macrophage and mannose receptor mediated cell uptake

#### GM-CSF stimulation

RAW 264.7 macrophage cells and HEK 293T human embryonic kidney cells were seeded at a density of 3 × 10^4^ cells per well in a 12-well plate and cultured overnight in complete Dulbecco’s modified Eagle’s medium (DMEM) in the presence and absence of GM-CSF (50 ng/ml). Next, the culture medium was replaced with fresh medium and treated with rhodamine, LR, and LRM at a concentration of 3 μg/ml. Cells were incubated for an additional 12 hours at 37°C. Cells were then washed and fixed with 4% paraformaldehyde as described above (see the Supplementary Materials). Fluorescence imaging was performed using a Keyence BZ-X800 fluorescence microscope (KEYENCE, IL, USA) to assess and compare cellular uptake across both cell lines.

#### *Cell uptake after blocking the mannose receptor* (*CD206*)

RAW 264.7 macrophage cells were seeded at a density of 3 × 10^4^ cells per well in a 12-well plate and cultured overnight in complete DMEM. The following day, cells were treated with anti-CD206 antibody at a concentration of 0.5 μg/ml and incubated for an additional 10 to 12 hours at 37°C to allow for receptor engagement. After antibody incubation, the medium was replaced with fresh medium containing either free rhodamine dye or rhodamine-labeled liposomal formulation at a concentration of 3 μg/ml. Cells were incubated for 3, 6, 12, and 24 hours to evaluate time-dependent uptake. At each time point, cells were detached by trypsinization, collected by centrifugation at 1000*g* for 3 min, and washed several times with phosphate-buffered saline (PBS) to remove unbound dye or formulation. The cell pellet was fixed with 4% paraformaldehyde for 10 min and subsequently washed twice more with PBS. The fixed cell suspensions were mounted onto glass slides and covered with coverslips. Fluorescence signals were then observed and imaged using a fluorescence microscopy.

### Cellular uptake in PMA-differentiated THP-1 macrophages

THP-1 monocytes (5 × 10^4^ cells per well) were seeded in RPMI 1640 (10% fetal bovine serum and 1% penicillin-streptomycin) in 24-well plates and treated with PMA (100 ng/ml) and incubated for 72 hours at 37°C/5% CO_2_ to induce macrophage-like differentiation, as confirmed by morphological adherence (*48*). Cells were then treated with free rhodamine or liposomal-rhodamine (1 or 5 μg/ml rhodamine equivalent) for 3 hours. Next, media were aspirated, wells were washed thrice with PBS, and cells were fixed in 4% paraformaldehyde for 20 min at RT. After three PBS washes, nuclei were counterstained with Hoechst 33342 (5 μg/ml, 10 min), followed by three final PBS washes. Fluorescence was imaged using a Keyence BZ-X810 microscope and quantified as mean integrated intensity (region of interest normalized).

### Therapeutic effect in DSS-colitis model

#### Model development

Male C57BL/6 mice (6 weeks old) were used to establish a DSS-induced colitis model. Before DSS administration, mice were acclimatized for 2 to 3 days with standard drinking water provided via drinking bottles, replacing the automated water supply system used in the animal facility. To induce colitis, mice were given 3.5% (w/v) DSS (molecular weight, 36,000 to 50,000 Da) in drinking water for 7 consecutive days. Throughout the treatment period, clinical signs of colitis were monitored daily. Parameters included body weight changes, stool consistency, the presence of occult or gross blood using a hemoccult test, and the calculation of DAI scores. Two mice were excluded before treatment allocation because, on day 5 following DSS administration, they did not develop the characteristic body weight loss and clinical signs observed in the rest of the cohort. These animals therefore did not meet the predefined inclusion criteria for successful colitis induction. Excluding them before randomization ensured comparable baseline disease severity and similar DAI scores across all treatment groups (Lipo-tm-HA, free tofacitinib, and controls), thereby improving the internal validity and interpretability of the therapeutic comparisons.

#### Treatment

Following induction of colitis with 3.5% DSS in drinking water as described above, mice were randomly assigned into six experimental groups (*n* = 5 per group) to evaluate the therapeutic efficacy of free and liposomal formulations of tofacitinib. The groups were as follows: (i) healthy control: no DSS administration and no drug treatment; (ii) DSS control: DSS administration only, no drug treatment; (iii) single-dose tofacitinib (10 mg/kg): colitis mice treated with a single oral dose of free tofacitinib; (iv) daily tofacitinib (5 mg/kg): colitis mice treated daily with oral tofacitinib at 5 mg/kg; (v) daily tofacitinib (10 mg/kg): colitis mice treated daily with oral tofacitinib at 10 mg/kg; and (vi) single-dose Lipo-tm-HA (10 mg/kg): colitis mice treated with a single oral dose of liposomal tofacitinib formulation (Lipo-tm-HA) at 10 mg/kg. Drug administration was initiated on day 5 of DSS exposure, and treatment continued on the basis of the respective dosing regimen. The study endpoint was day 10 post–DSS initiation. Throughout the study, clinical parameters of colitis were monitored to calculate DAI and assess therapeutic efficacy.

#### Therapeutic efficacy evaluation

To evaluate the therapeutic efficacy, we monitored the DAI scores. It defined as follows: (i) weight loss: 0 (no loss), 1 (1 to 5%), 2 (5 to 10%), 3 (10 to 20%), and 4 (>20%); (ii) stool consistency: 0 (normal), 2 (loose stool), and 4 (diarrhea); and (iii) bleeding: 0 (no blood), 1 (hemoccult positive), 2 (hemoccult positive with visible pellet bleeding), and 4 (gross bleeding with perianal hemorrhage). At the study endpoint (day 10), mice were euthanized, and colon length was measured. Additionally, spleens were excised and weighed to assess systemic inflammatory responses. These physiological and anatomical parameters were used in conjunction with DAI scores to compare the efficacy of different treatment regimens.

#### Percentage mucosal injury in the colon

To assess the extent of mucosal injury following treatment with tofacitinib and Lipo-tm-HA, colonic tissues were processed using the Swiss roll technique. Briefly, freshly excised colons were gently flushed with PBS, rolled longitudinally, and fixed in 10% neutral-buffered formalin for histological analysis. Fixed tissues were embedded in paraffin blocks, sectioned, and stained with H&E by the Reference Histology Core, Department of Pathology, Johns Hopkins University (reference nos. CF-12103484-3 and CF-12104480-0). The stained slides were submitted to the core facility for high-resolution scanning at ×20 and ×40 magnification. The percentage of mucosal injury in each sample was evaluated on the basis of the histopathologic features described in (*49*). Colonic mucosa was deemed to be injured if one of the following was present: (i) moderate to severe inflammation; (ii) intraepithelial lymphocytosis, erosion, or ulceration; or (iii) epithelial hyperplasia, dysplasia, or neoplasia.

### Biodistribution study for colon-targeted delivery by IVIS

For biodistribution analysis, colitis was induced in male C57BL/6 mice using 3.5% (w/v) DSS in drinking water for 7 days, as previously described. On day 8, mice were orally administered either free rhodamine dye or rhodamine-labeled liposomal formulation (Lipo-rm-HA) at a dose of 2.5 mg/kg (rhodamine equivalent). After 24 hours postadministration, blood samples were collected via inferior vena cava under anesthesia, and mice were perfused with PBS to eliminate residual blood. Major organs, including the heart, lungs, liver, spleen, kidneys, small intestine, and colon, were harvested. The colon and small intestine were flushed three to four times with PBS to remove internal contents. Collected tissues were subjected to ex vivo fluorescence imaging using an IVIS (excitation, 500 nm; and emission, 540 nm) to detect rhodamine signal.

### Tissue distribution of Lipo-tm-HA by HPLC

Acute DSS-induced colitis was established in C57BL/6 mice by 7 days of 3% DSS in drinking water. Mice then received a single oral dose of Lipo-tm-HA or free tofacitinib (10 mg/kg). After 24 hours, mice were anesthetized; blood was collected via cardiac puncture; and tissues (heart, lung, liver, spleen, kidney, small intestine, and colon) were excised, snap frozen, and stored at −80°C until analysis.

Tissues (∼100 mg) were homogenized in PBS (100 mg/ml) using a tissue disruptor and centrifuged at 10,000*g* for 30 min at 4°C, and supernatants were collected. Plasma and tissue supernatants (100 μl) underwent liquid-liquid extraction as described (*50*): Briefly, samples were basified with 25 μl of 20% (v/v) ammonia, vortexed, and sonicated (30 s). *Tert*-butyl methyl ether (1.5 ml) was added, followed by vortexing (3 min), centrifugation (4000*g*, 10 min, 4°C), organic layer transfer, evaporation (SpeedVac at 50°C), and reconstitution in 100 μl of HPLC mobile phase. Tofacitinib was quantified by reverse-phase HPLC (C18 column, 9.5 mm by 250 mm, 5 μm; λ = 285 nm; flow rate, 1.0 ml/min) using gradient elution: (A) 10 mM ammonium acetate (pH 5.0, adjusted with acetic acid) and (B) acetonitrile. Gradient: 0 to 4.9 min (99:1, A:B), 5 to 10 min (98:2, A:B), and 10 to 15 min (95:5, A:B).

### Systemic exposure and pharmacokinetic study

To evaluate systemic exposure, C57BL/6 male mice were administered free rhodamine dye or Lipo-rm-HA orally at a dose of 2.5 mg/kg (rhodamine equivalent). Blood and stool samples were collected at predetermined time points: 30 min, 1 hour, 1.5 hours, 3 hours, 4.5 hours, 6 hours, 8 hours, and 24 hours postadministration. Samples were collected into individual wells of a black 24-well plate to minimize background fluorescence. Fluorescence intensity was measured using an IVIS with excitation and emission wavelengths set at 500 and 540 nm, respectively.

### Statistical analysis

All graphical illustrations and schematics were created using BioRender, Microsoft PowerPoint, and Microsoft Excel. Statistical analyses were performed using Microsoft Excel and BioRender, as appropriate. Data were tested for normality (Shapiro-Wilk test) and homogeneity of variance (Levene’s test) before applying parametric tests; when these assumptions were not met, appropriate nonparametric tests were used. Comparisons between two groups were conducted using unpaired two-tailed Student’s *t* tests. For comparisons among multiple groups, one-way or two-way analysis of variance (ANOVA) was used, followed by Bonferroni’s post hoc *t* test for pairwise comparisons. Data are presented as means ± SD, unless otherwise specified. A *P* value of less than 0.05 was considered statistically significant. For all experiments, *n* ≥ 3 or 5 samples per group unless otherwise indicated.
