# Supplementary material for: An innovative colon inflammation-targeting platform for ulcerative colitis treatment
Source: Sci Adv. 2026 Oct 23;12(43):eaeb1963. doi: 10.1126/sciadv.aeb1963 (PMC13693676; doi:10.1126/sciadv.aeb1963)
Supplement: Supplementary file 1 [file sciadv.aeb1963_sm.pdf]

Science Advances
AAAS

Supplementary Materials for

An innovative colon inflammation-targeting platform for ulcerative colitis treatment

Waliul Islam *et al.*

Corresponding author: Florin M. Selaru, fselaru1@jhmi.edu

Sci. Adv. **12**, eaeb1963 (2026)
DOI: 10.1126/sciadv.aeb1963

This PDF file includes:

Supplementary Materials and Methods
Table S1
Figs. S1 to S17

MATERIALS AND METHODS

Chemicals

Tofacitinib was procured from Medkoo Biosciences Inc. (NC, USA). Distearoylphosphatidylcholine (DSPC) was obtained from Polysciences Inc. (PA, USA). D-mannitol, mannose, cholesterol, hyaluronic acid, sodium tetraborate, sodium hydride, chloroform, phosphotungstic acid, lipopolysaccharide (LPS), granulocyte-macrophage colony-stimulating factor (GM-CSF), monoclonal anti-CD206 antibody, protease inhibitor cocktail and human transferrin were all purchased from Millipore Sigma-Aldrich (MO, USA). Phorbol 12-myristate 13-acetate were purchased from Medchemexpress, NJ, USA. ELISA kits for TNF-α, IL-6, IL-1β were obtained from RayBiotech, GA, USA. Rhodamine-conjugated transferrin was acquired from Antibodies Online (PA, USA), and Rhodamine 123, dextran sodium sulfate (DSS, catalog# J63606.22) MTT reagent, and ProLong™ Gold Antifade Mountant were purchased from Life Technologies Corporation (CA, USA). Isoflurane was sourced from Jeffers Inc. (AL, USA). Simulated intestinal fluid was obtained from Chemazone Inc. Catalog# 7732-18-6 (Canada), and simulated gastric juice was acquired from Carolina Biological Supply, item# 864603 (NC, USA). Tissue-Tek® OCT compound was procured from Sakura Finetek Inc. (CA, USA), and Cell Reservoir One was obtained from Nacalai Inc. (CA, USA). All other reagents and solvents used in this study were of analytical or reagent grade and were used as received from commercial suppliers without further purification.

Cells and animals

RAW 264.7 macrophage, THP-1 and human embryonic kidney (HEK293T) cells were maintained and cultured in vitro by using Dulbecco's Modified Eagle's Medium (Millipore Sigma-Aldrich, MO, USA) supplemented with 10% fetal bovine serum (FBS) (Biosera, Kansas City, MO, USA) and 1% penicillin streptomycin antibiotics (Nacalai Inc) under 5% CO_2/air at 37 °C. C57BL/6, male mice, all 6 weeks old, were purchased from the Jackson Laboratory, ME, USA.

Preparation of fluorescently labeled liposomal formulation

Fluorescently labeled liposomes were prepared using the same procedure of Lipo-tm-HA formulation, incorporating 4-5% (wt) of rhodamine 123 into the lipid mixture during the thin-film formation 2

step. The formulation was subjected to identical steps of hydration, extrusion, dialysis, and HA coating, as described, to generate a fluorescent analog suitable for tracking and biodistribution studies.

Characterization of liposomal tofacitinib-mannitol coated with hyaluronic acid (Lipo-tm-HA)

1. *UV-vis spectroscopy*

Liposomal formulations, including both rhodamine 123-labeled and unlabeled variants, were dissolved in deionized water to a final concentration of 0.05 mg/mL. UV-vis spectra of the samples were recorded using a DU® 800 spectrophotometer (Beckman Coulter, CA, USA). Drug loading was quantified by comparing the absorbance values to standard calibration curves prepared for tofacitinib and rhodamine 123.

2. *High performance liquid chromatography (HPLC)*

Lipo-tm-HA was dissolved in phosphate-buffered saline (PBS) at a concentration of 5 mg/mL. Size exclusion chromatography (SEC) was performed using an Agilent Technologies HPLC system (Agilent Technologies, Inc., DE, USA) equipped with a Shodex Asahipak GF-310 HQ column (7.5 × 300 mm) and a photodiode array detector set at 285 nm. The mobile phase consisted of PBS (pH 7.0), delivered at a constant flow rate of 0.8 mL/min.

Binding of Lipo-tm-HA to transferrin

1. *Detection of fluorescently labeled transferrin by Fluorescence spectroscopy and IVIS*

Rhodamine-labeled transferrin was co-incubated with Lipo-tm-HA at various weight ratios and incubated at 37 °C for 30 minutes to allow surface association. Following incubation, bound and unbound transferrin were separated by ultracentrifugation as previously described. The liposomal pellet was washed five times with deionized water to remove unbound transferrin. Fluorescence intensity of rhodamine in both the liposomal pellet and the corresponding supernatants was measured using a fluorescence spectrophotometer to quantify the extent of transferrin binding. Additionally, the rhodamine signal associated with the liposomal formulations was visualized using fluorescence imaging with an in vivo imaging system (IVIS).

2. *Size exclusion chromatography (SEC) by HPLC*

Lipo-tm-HA was mixed with transferrin at a weight ratio of 1:3 and incubated at 37 °C for 30 minutes to allow interaction. Following incubation, size exclusion chromatography (SEC) was conducted using an Agilent Technologies HPLC system (Agilent Technologies, Inc., DE, USA) equipped with a Shodex Asahipak GF-310 HQ column (7.5 × 300 mm) and a photodiode array detector set at 280 nm and 285 nm. The mobile phase consisted of phosphate-buffered saline (PBS, pH 7.0), delivered at a constant flow rate of 0.8 mL/min. This analysis enabled the evaluation of transferrin binding to the liposomal surface based on elution profiles and absorbance signals.

3. *UV-vis spectroscopy*

Lipo-tm-HA was co-incubated with transferrin at weight ratios of 1:1 and 1:3 at 37 ℃ for 30 minutes to assess potential interactions. Following incubation, changes in the spectral profile were monitored using UV–vis spectroscopy as previously described. Spectral shifts and absorbance variations were analyzed to evaluate the extent of interaction between transferrin and the liposomal formulation.

Macrophage and mannose receptor mediated cell uptake

1. *Cell uptake in dose dependent manner*

RAW 264.7 macrophage cells were seeded at a density of 3×10^4 cells per well in a 12-well plate and cultured overnight in Dulbecco's Modified Eagle Medium (D-MEM) supplemented with 10% fetal bovine serum (FBS) and 1% penicillin-streptomycin. The following day, the culture medium was replaced with fresh medium containing either free rhodamine dye, rhodamine-labeled liposomal formulation (LR), or mannitol-derived rhodamine-labeled liposomal formulation (LRM) at concentrations of 1, 3, and 5 µg/mL. Cells were incubated for an additional 12 hours at 37 °C. After incubation, cells were washed twice with phosphate-buffered saline (PBS) and fixed with 4% paraformaldehyde for 15-20 minutes at room temperature. The fixed cells were then washed three times with PBS. Fluorescence signals were subsequently analyzed using a Keyence BZ-X800 fluorescence microscope (KEYENCE, IL, USA) to assess cellular uptake.

2. *Cell uptake in the presence of lipopolysaccharide (LPS) and mannose*

RAW 264.7 macrophages were seeded in 12-well plates at 3 × 10^4 cells/well in complete DMEM and allowed to adhere overnight at 37 °C in a humidified 5% CO_2 incubator. The following day, cells were stimulated with LPS (5 µg/mL) to induce macrophage activation and with mannose (5 or 10 mM) to see its modulation effect. After 12 h, cells were washed with PBS and subsequently treated with LRM for an additional 6 h at 37 °C. Cells were then fixed with 4% paraformaldehyde for 10 min at room temperature, washed with PBS, and subjected to fluorescence imaging using a fluorescence microscope.

Therapeutic effect of Lipo-tm-HA in DSS-colitis model

1. *Measurement of proinflammatory cytokines by ELISA*

DSS-induced colitis was induced in C57BL/6 mice as described previously. At day 5 post-DSS initiation, mice received a single oral dose of Lipo-tm-HA (10 mg/kg), and equimolar free tofacitinib (10 mg/kg/day, p.o.) administered daily for 5 consecutive days. On day 10, mice were euthanized humanely, and colons were excised, flushed 4–5 times with ice-cold PBS to remove luminal contents, placed in ice-cold PBS. Colon segments (~100 mg) were homogenized in ice-cold PBS (100 mg tissue/mL) using a tissue disruptor, during homogenization a protease inhibitor cocktail (1:1000 dilution) was added. Next, the tissue homogenates were centrifuged at 10,000 × g for 10 min at 4°C, and supernatants collected and stored at −80°C until analysis. TNF-α, IL-6, and IL-1β concentrations were quantified in triplicate using RayBiotech Mouse ELISA kits according to the manufacturer's protocol. Absorbance was measured on a microplate reader at 450 nm, with cytokine levels determined from standard curves.

2. *Measurement of CD45+ and macrophage (M1/M2) populations*

An acute DSS colitis model was established in C57BL/6 mice as described previously. On day 5, Lipo-tm-HA was administered orally at a dose of 10 mg/kg. On day 10, mice were euthanized and the colon tissues were harvested. The luminal contents were gently removed by flushing the colon with ice-cold PBS, and the tissues were kept on ice in PBS until processing. The colons were then minced into small pieces and digested with appropriate enzymes at 37°C for 2 h with gentle agitation until a single-cell suspension was obtained. The enzymatic reaction was stopped by adding DMEM supplemented with 10% FBS. The

resulting cell suspension was washed three to four times with ice-cold PBS by centrifugation at 300 × g for 5 min at 4°C. Cell viability was assessed before staining. CD45+ leukocytes and M1/M2 macrophage populations were then analyzed by flow cytometry.

Biodistribution study for colon targeted delivery

Drug localization in different tissue by fluorescence microscopy

Following the ex vivo biodistribution study, harvested colon and other major tissues were fixed in 10% neutral-buffered formalin for at least 48 hours at room temperature. Fixed tissues were then embedded in optimal cutting temperature (O.C.T.) compound and cryosectioned into 10 μm thick slices using a cryostat. Sections were mounted onto glass microscope slides. Slides were washed five times with 1× phosphate-buffered saline (PBS) to remove excess O.C.T. compound. A drop of ProLong™ Gold Antifade Mountant was applied to each tissue section before being covered with a glass coverslip. Fluorescence signals were then visualized using a Keyence BZ-X800 fluorescence microscope.

Systemic exposure and pharmacokinetic study

Fluorescence plate reader

For quantification of drug concentration in blood, approximately 400 μL of ethanol was added to each well containing the collected blood samples to facilitate extraction of the rhodamine-labeled compound. The ethanol-blood mixture was then transferred to microcentrifuge tubes and vigorously vortexed for 1–2 minutes to ensure thorough mixing and drug extraction. Samples were centrifuged at 12,000 × g for 15 minutes at room temperature. The resulting supernatant was carefully collected and transferred to a black 96-well plate. Fluorescence intensity was measured using a fluorescence plate reader (excitation: 490 nm, emission: 540 nm) to determine the concentration of rhodamine-labeled drug in the blood.

Toxicity study of Lipo-tm-HA

1. *In vitro toxicity*

The cytotoxicity of free tofacitinib and Lipo-tm-HA was evaluated using the MTT assay in RAW264.7 macrophage cells and HEK 293T human kidney cells. Cells were seeded in 96-well culture plates at a density of 1 × 10^4 cells/well and incubated overnight in Dulbecco's Modified Eagle Medium (DMEM)

supplemented with 10% fetal bovine serum (FBS), 100 U/mL penicillin, and 100 μg/mL streptomycin under standard culture conditions (37 °C, 5% CO_2, 95% air). After overnight incubation, the culture medium was replaced with fresh medium containing various concentrations of either free tofacitinib or Lipo-tm-HA. Cells were incubated for 24 hours at 37 °C. Subsequently, 20 μL of MTT solution (5 mg/mL in PBS) was added to each well and incubated for an additional 4 hours. Formazan crystals formed by viable cells were dissolved in 100 μL of DMSO, and absorbance was measured at 570 nm using a microplate reader. Cell viability was expressed as a percentage relative to untreated control cells.

2. *In vivo toxicity*

In vivo toxicity of free tofacitinib and Lipo-tm-HA was evaluated in male C57BL/6 mice. Mice were randomly assigned to receive either free tofacitinib or Lipo-tm-HA, both administered orally (PO) at a dose of 10 mg/kg. Free tofacitinib was given daily for 5 consecutive days, while Lipo-tm-HA was administered as a single dose to mimic the treatment regimen used for therapeutic efficacy studies. On day 5, mice were euthanized, and major organs—including the heart, lungs, liver, spleen, and kidneys—were harvested and fixed in 10% formalin. Histological analysis was performed by hematoxylin and eosin (H&E) staining at the Reference Histology Core Facility, Department of Pathology, Johns Hopkins University. The histopathological evaluations were conducted by a pathologist to assess potential signs of organ toxicity.

Supplementary Table

Sup. table 1. Physicochemical properties of different liposomal formulations.

Drug name	Hydrodynamic diameter by DLS (nm)	Particle size by TEM (nm)	Surface charge (mV)	Polydispersity index (PDI)	Drug loading (%)
Lipo-t	170 ± 20	120 ± 10	- 0.02	0.04	10-12
Lipo-tm	185 ± 10	120 ± 10	-18	0.042	10-12
Lipo-tm-HA	280 ± 20	130 ± 20	-32	0.056	10-12
Lipo-rm-HA	320 ± 20			0.045	2-3
Hyaluronic acid	338 ± 10	150-200	- 38	0.4	

Lipo-t: Liposomal tofacitinib, **Lipo-tm:** Liposomal formulation with tofacitinib and mannitol, **Lipo-tm-HA:** Liposomal formulation with tofacitinib and mannitol coated with hyaluronic acid, **Lipo-rm-HA:** Liposomal formulation with rhodamine and mannitol coated with hyaluronic acid

Supplementary figures:

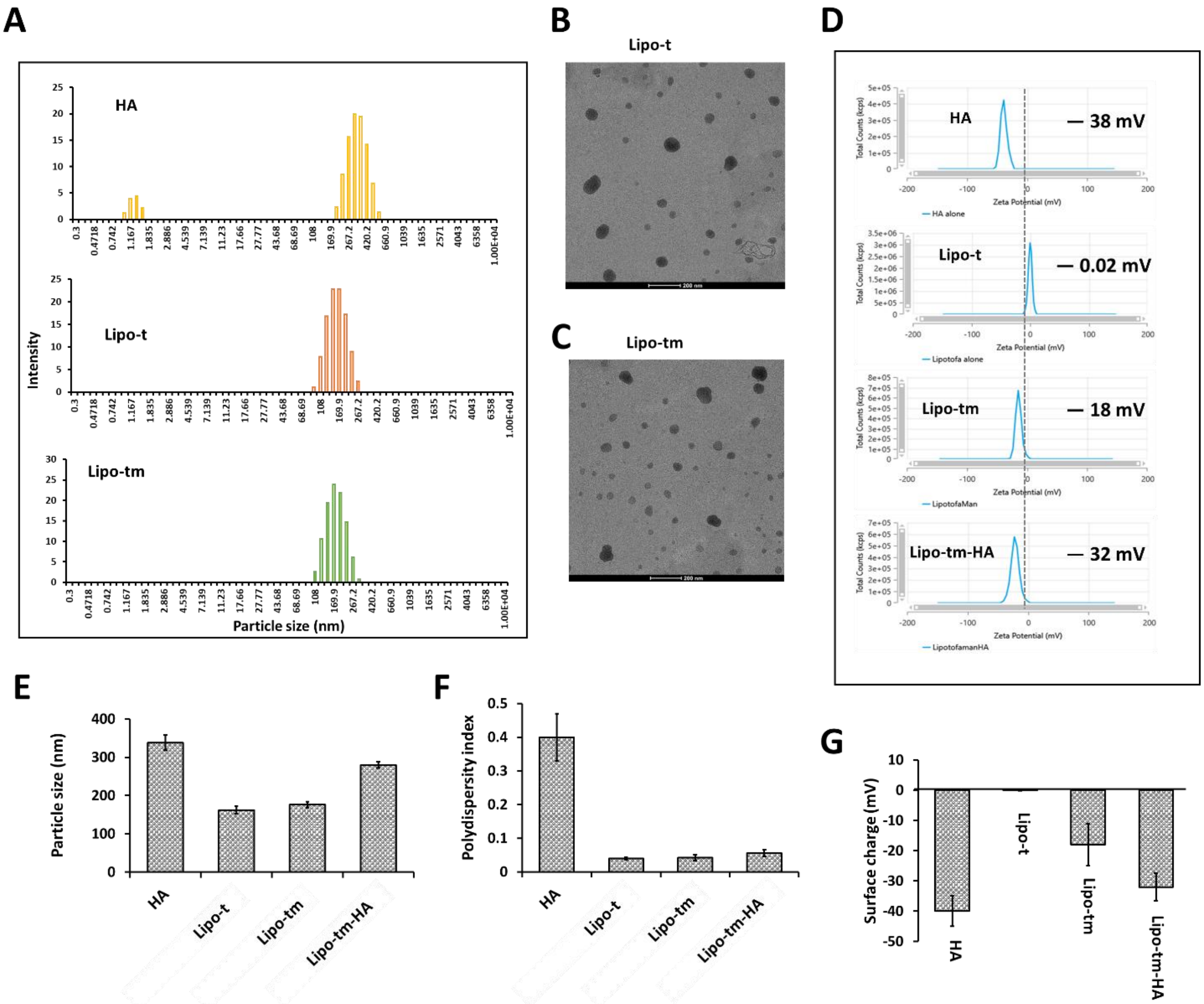

Fig. S1. Physicochemical properties of different liposomal formulation. (A) Hydrodynamic diameter of hyaluronic acid (HA), liposome-encapsulated tofacitinib (Lipo-t), and mannitol-engineered liposomal tofacitinib (Lipo-tm) measured by DLS in deionized water (DW), showing distinct particle size profiles. **(B)** Representative TEM image of Lipo-t, revealing the particle morphology and size distribution (scale bar = 200 nm). **(C)** TEM image of Lipo-tm after staining with 0.01% phosphotungstic acid, confirming the particle size and structural integrity (scale bar = 200 nm). **(D)** Surface charge (zeta potential) of different liposomal formulations, including HA (-38 mV), Lipo-t (-0.02 mV), Lipo-tm (-18 mV), and Lipo-tm-HA (-32 mV), demonstrating charge modulation upon mannitol fabrication and HA coating. **(E)** Hydrodynamic diameter of various liposomal formulations, indicating a slight increase in particle size following HA coating. **(F)** Polydispersity index (PDI) of the different formulations, highlighting the uniformity of the liposome populations. **(G)** Zeta potential measurements of each formulation in DW, confirming the surface charge changes associated with HA coating and mannitol modification. Data are presented as mean ± SD (n = 3).

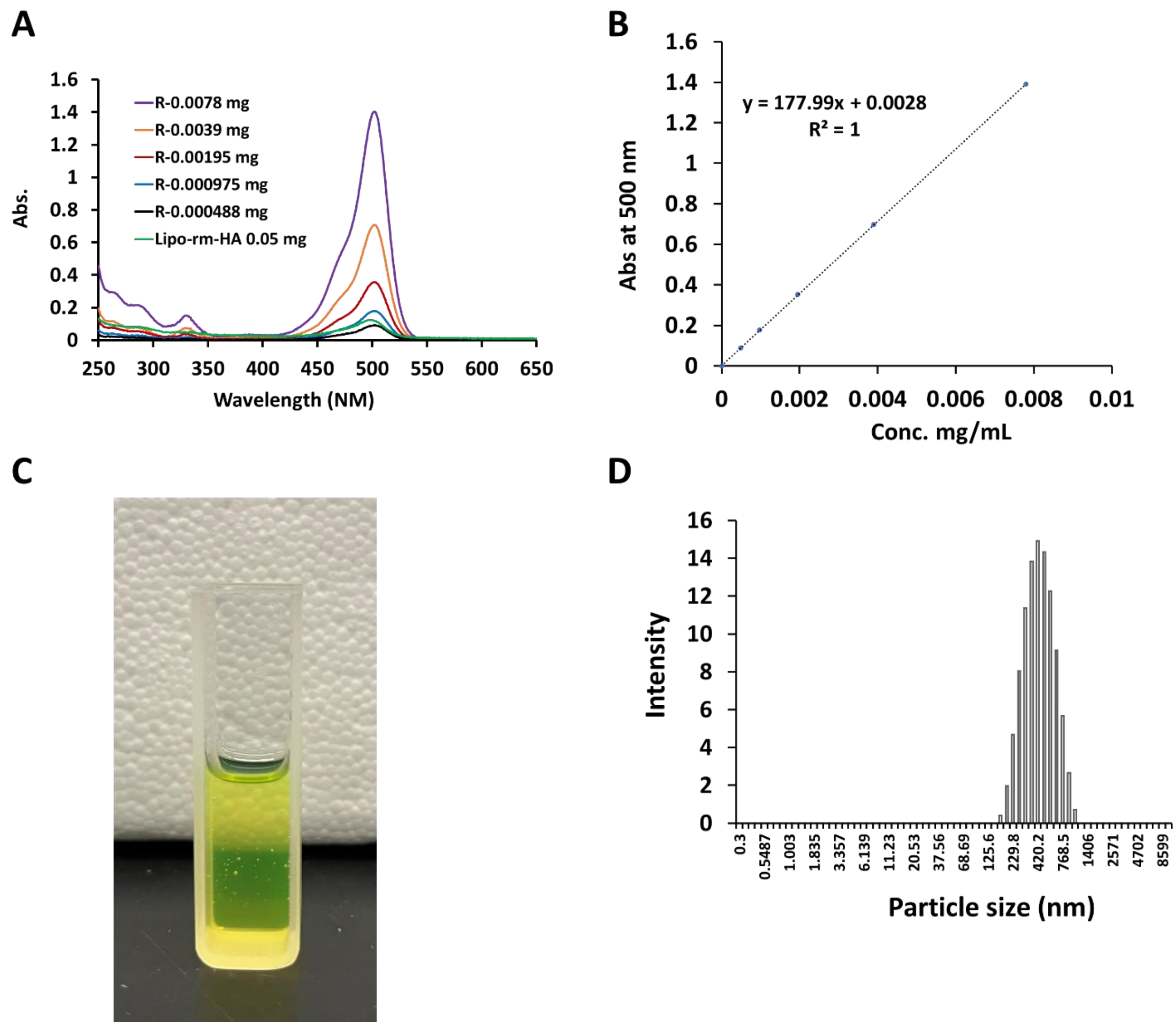

Fig. S2. Characterization of rhodamine labeled liposomal formulation (Lipo-rm-HA). (A) UV-vis absorption spectra of free rhodamine at varying concentrations alongside Lipo-rm-HA at 0.05 mg/mL in water, confirming the spectral profile of the liposomal formulation. **(B)** Standard curve of free rhodamine, generated at its maximum absorbance wavelength of 510 nm, used for quantification of rhodamine content in the liposomal formulation. **(C)** Representative image of rhodamine-labeled Lipo-rm-HA dispersed in water, demonstrating the homogeneous dispersion of the fluorescent formulation. **(D)** Hydrodynamic diameter of Lipo-rm-HA in water, measured by DLS, indicating the size distribution and stability of the liposomal formulation.

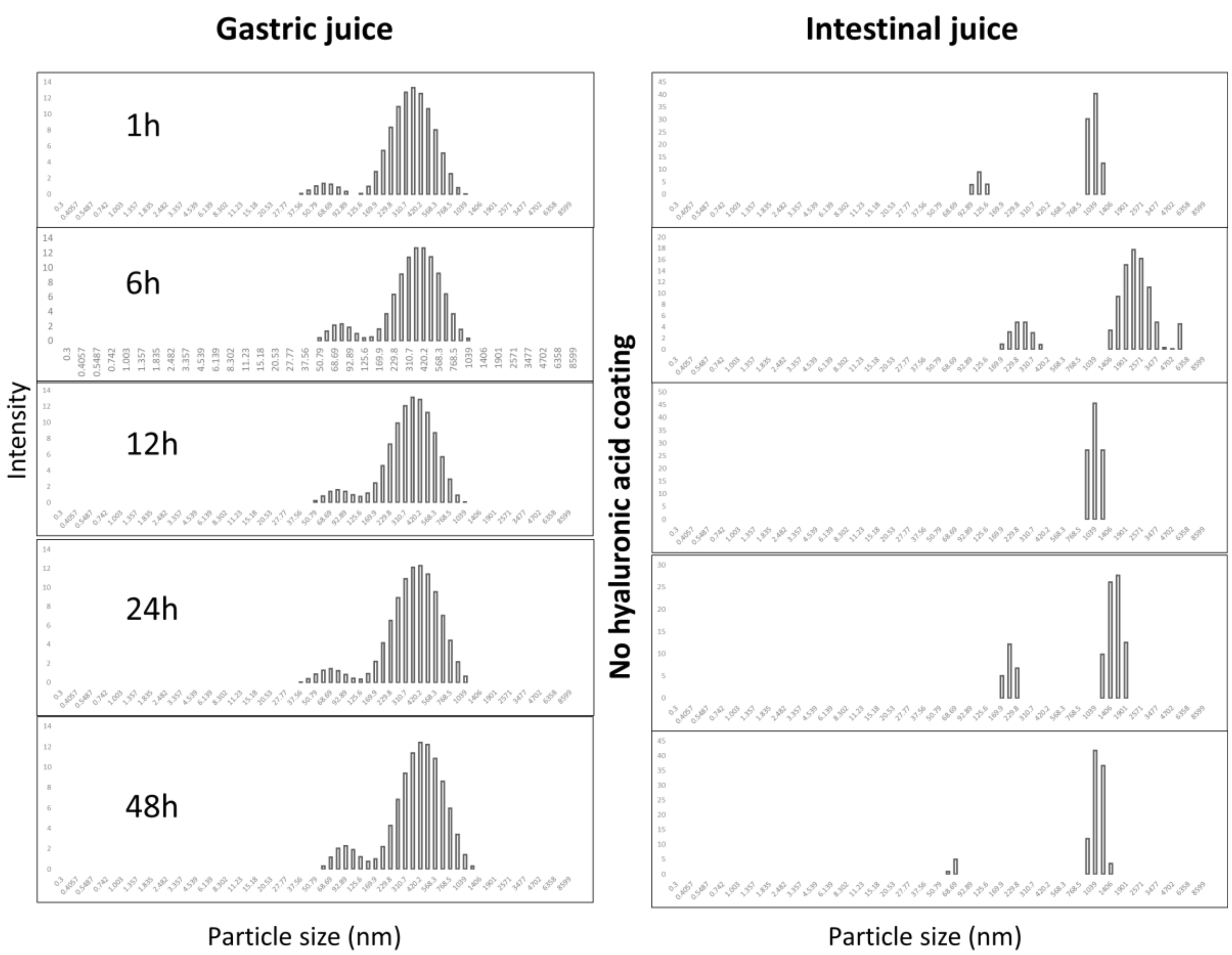

Fig. S3. Stability of non-hyaluronic acid coated liposomal formulation. (A) Stability assessment of the non-HA-coated liposomal formulation in simulated gastric juice, evaluated by dynamic light scattering (DLS). The absence of a single defined peak indicates instability or disassembly in the gastric environment, highlighting the critical role of HA coating in preserving structural integrity. **(B)** Similarly, in simulated intestinal juice, the non-coated liposomes exhibited multiple peaks in the DLS profile, suggesting poor stability. This underscores the protective effect of HA coating in enhancing the formulation's stability under gastrointestinal conditions.

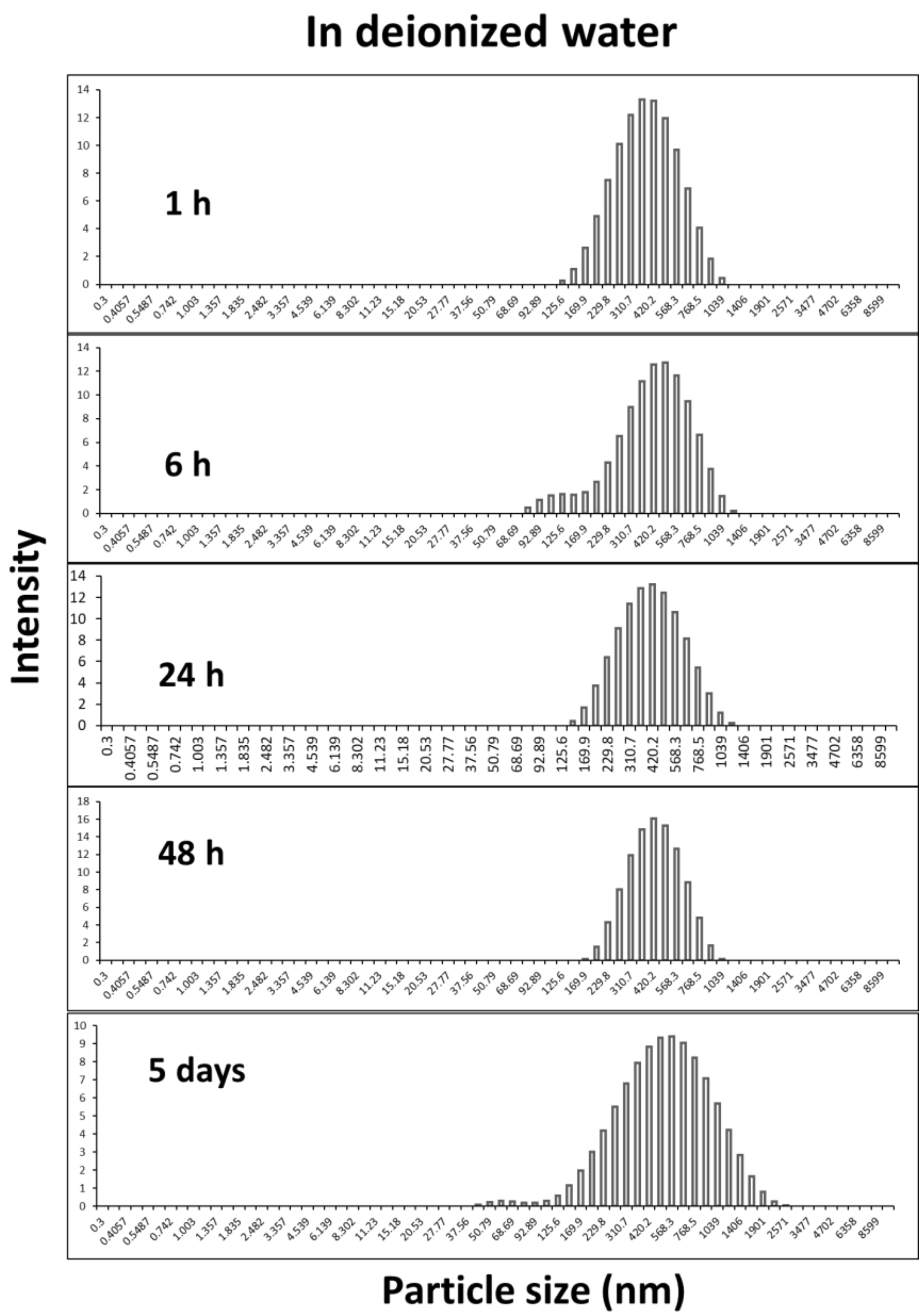

Fig. S4. Stability of Lipo-tm-HA in deionized water (DW). The Lipo-tm-HA formulation exhibited stability in DW, as indicated by a well-defined peak in the dynamic light scattering (DLS) profile. However, prolonged incubation for 5 days resulted in a broader peak, suggesting partial aggregation or structural alterations over time. These findings indicate that while Lipo-tm-HA remains stable in DW for short-term storage, extended incubation may compromise its uniformity and stability.

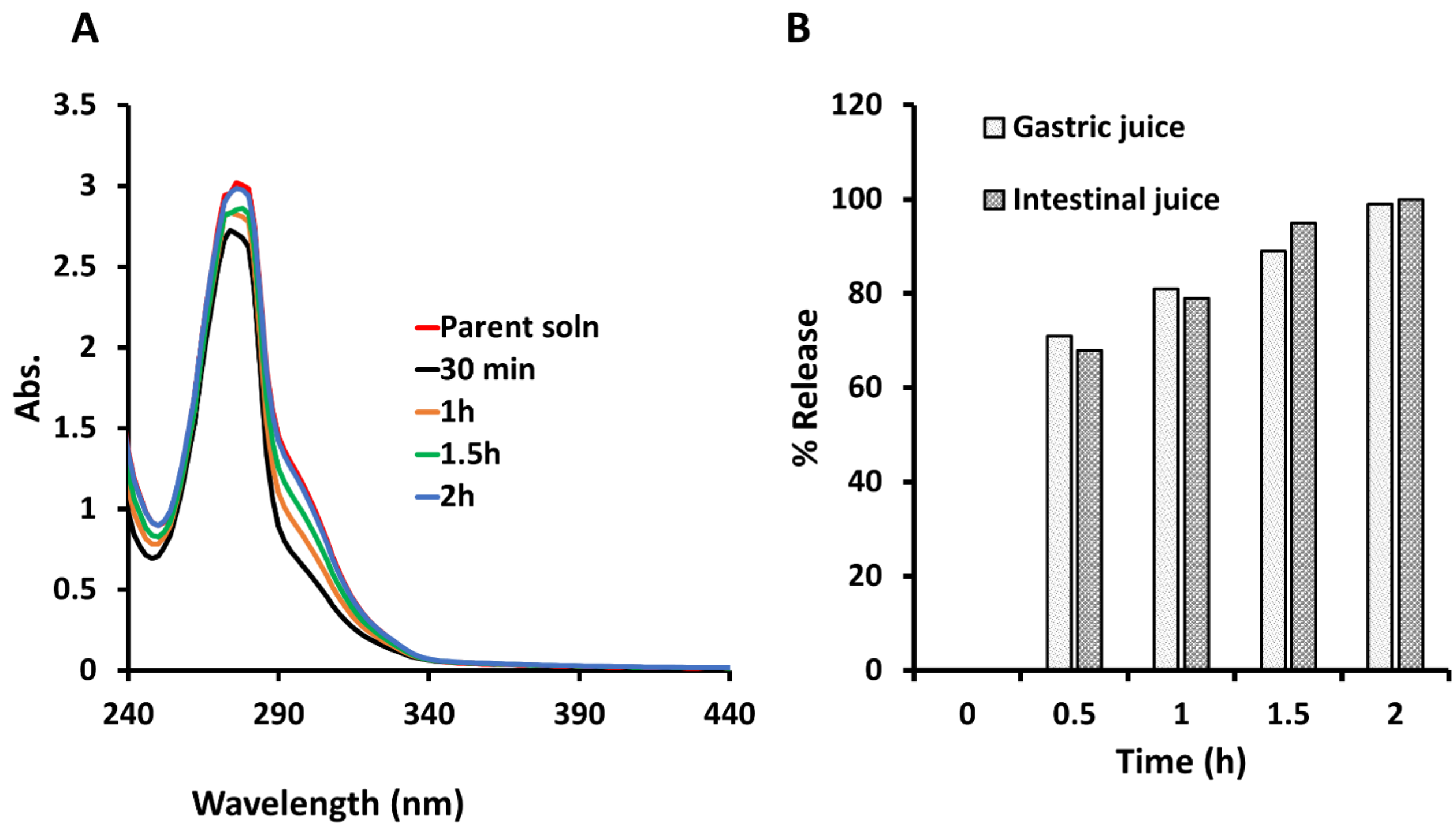

Fig. S5. In vitro release of tofacitinib in simulated gastric and intestinal fluids using a Visking dialysis tube. Tofacitinib (free drug) was loaded into Visking tubing and incubated in simulated gastric or intestinal juice at 37°C under gentle agitation. **(A)** Representative UV–visible absorption spectra of the external medium at different time points, showing the tofacitinib absorbance peak outside the dialysis tube. **(B)** Cumulative percentage of tofacitinib released into the external medium over time in simulated gastric and intestinal juice, demonstrating that free tofacitinib release reached a plateau within 1 h of incubation at 37°C.

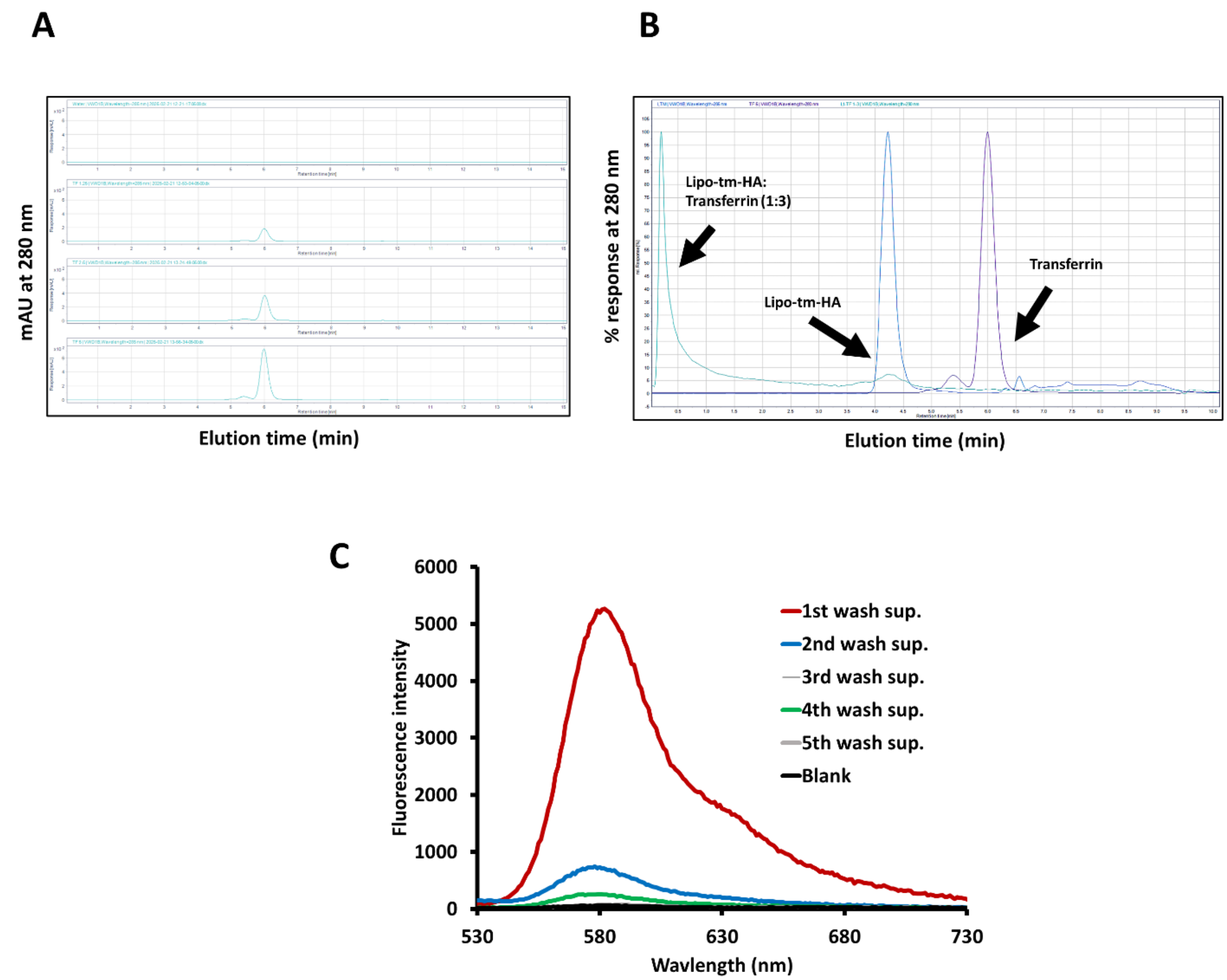

Fig. S6. Binding of Lipo-tm-HA to transferrin. (A) Size exclusion chromatography (SEC) of transferrin standards at varying concentrations, performed using an Agilent HPLC system equipped with a Shodex GF-310 HQ column (7.5 mm × 300 mm) and photodiode array detection at 280 nm. The mobile phase consisted of phosphate-buffered saline (PBS) with a flow rate of 1 mL/min. **(B)** SEC analysis of transferrin, Lipo-tm-HA, and the Lipo-tm-HA-transferrin complex. The complex exhibited a distinct peak in the void volume, indicating the formation of a larger molecular complex. In contrast, free transferrin eluted at 6.5 min, while Lipo-tm-HA alone eluted at 4.5 min. The black arrows denote the elution positions of the individual and complexed components, highlighting the successful formation of the Lipo-tm-HA-transferrin complex. **(C)** Fluorescence signal detection in the supernatant after ultra-centrifugal separation, assessed by fluorescence spectroscopy. Rhodamine-labeled transferrin was incubated with Lipo-tm-HA, followed by ultracentrifugation to isolate the liposome-transferrin complex. After five consecutive washes, no detectable fluorescence signal was observed in the supernatant

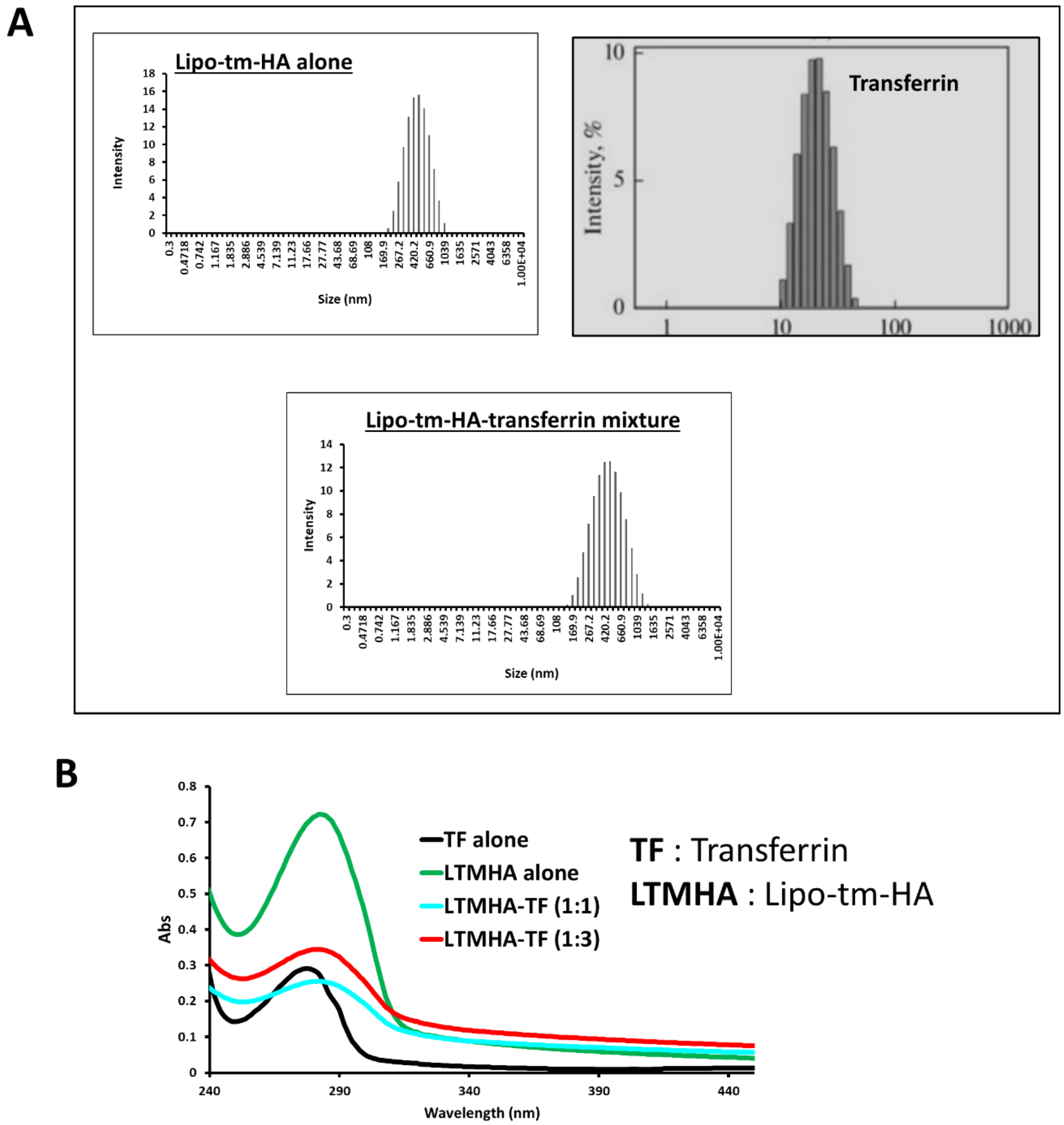

Fig. S7. Binding transferrin to Lipo-tm-HA by DLS and UV-vis spectroscopy. (A) Hydrodynamic diameter analysis of Lipo-tm-HA (~300 nm), transferrin (~10 nm), and the Lipo-tm-HA–transferrin complex (~300 nm) by dynamic light scattering (DLS). No significant change in the hydrodynamic diameter of Lipo-tm-HA was observed after complexation with transferrin, suggesting that transferrin attachment does not substantially alter the particle size. **(B)** UV-vis spectra of transferrin, Lipo-tm-HA, and their mixtures at varying ratios. While no significant shift in the characteristic absorption peaks was detected, the mixture exhibited a slight broadening of the peak between 260–290 nm, indicating subtle intermolecular interactions between transferrin and the liposomal formulation.

Lipo-rm with GM-CSF **Lipo-rm after blocking with CD206**

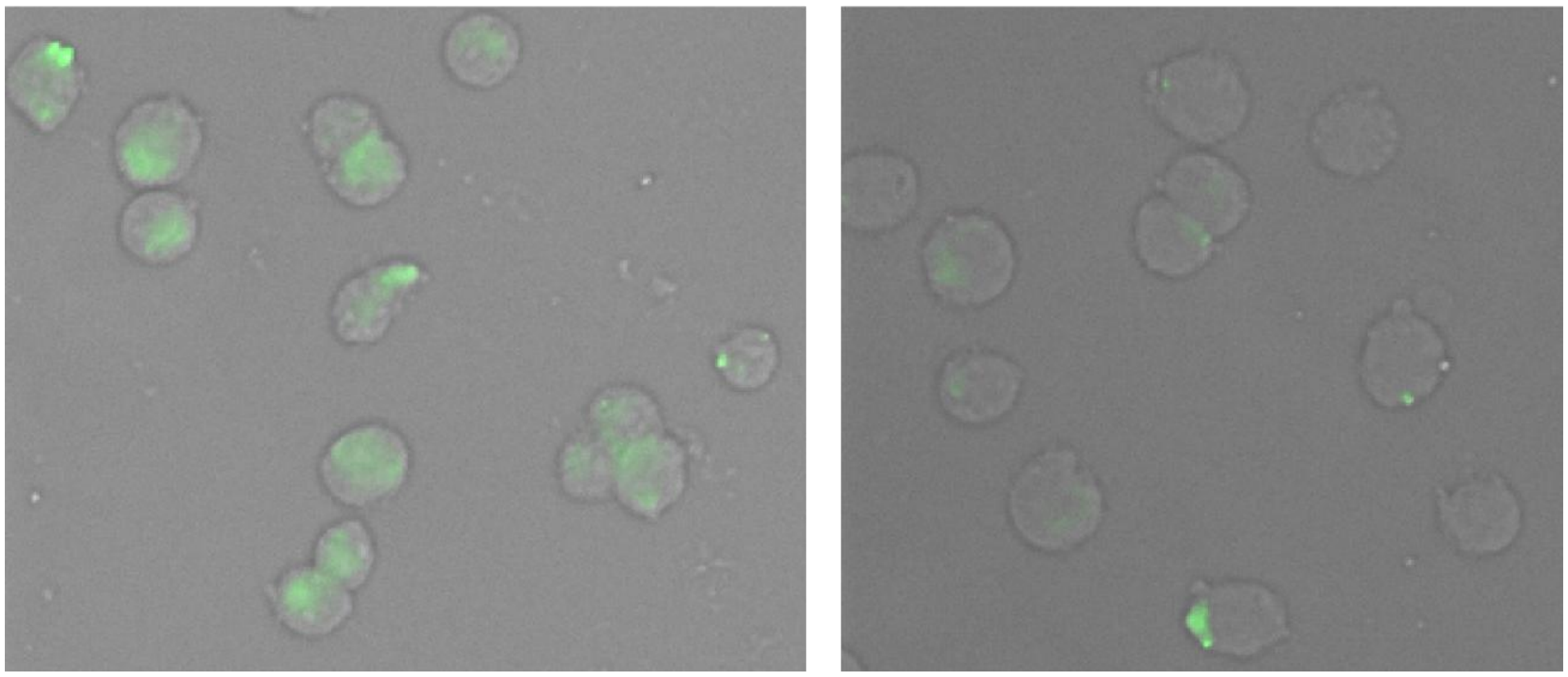

Fig. S8. Localization of rhodamine labeled liposomal formulation (Lipo-rm) in RAW 264.7 cells. Cellular internalization of Lipo-rm in RAW264.7 macrophages stimulated with GM-CSF (50 ng/mL) for 12 hours at 37°C examined by fluorescence microscopy. The enhanced intracellular fluorescence signal indicates increased drug uptake, suggesting receptor-mediated or endocytosis-driven internalization of the mannitol-engineered liposomal formulation (**left image**). Lipo-rm uptake in macrophages pre-treated with mannose receptor (CD206) blocking antibody (0.5 µg/mL) for 6 hours prior to drug incubation. The reduced intracellular fluorescence signal after blocking confirms that mannose receptor-mediated endocytosis plays a key role in the cellular uptake of the mannitol-functionalized liposomal formulation (**right image**).

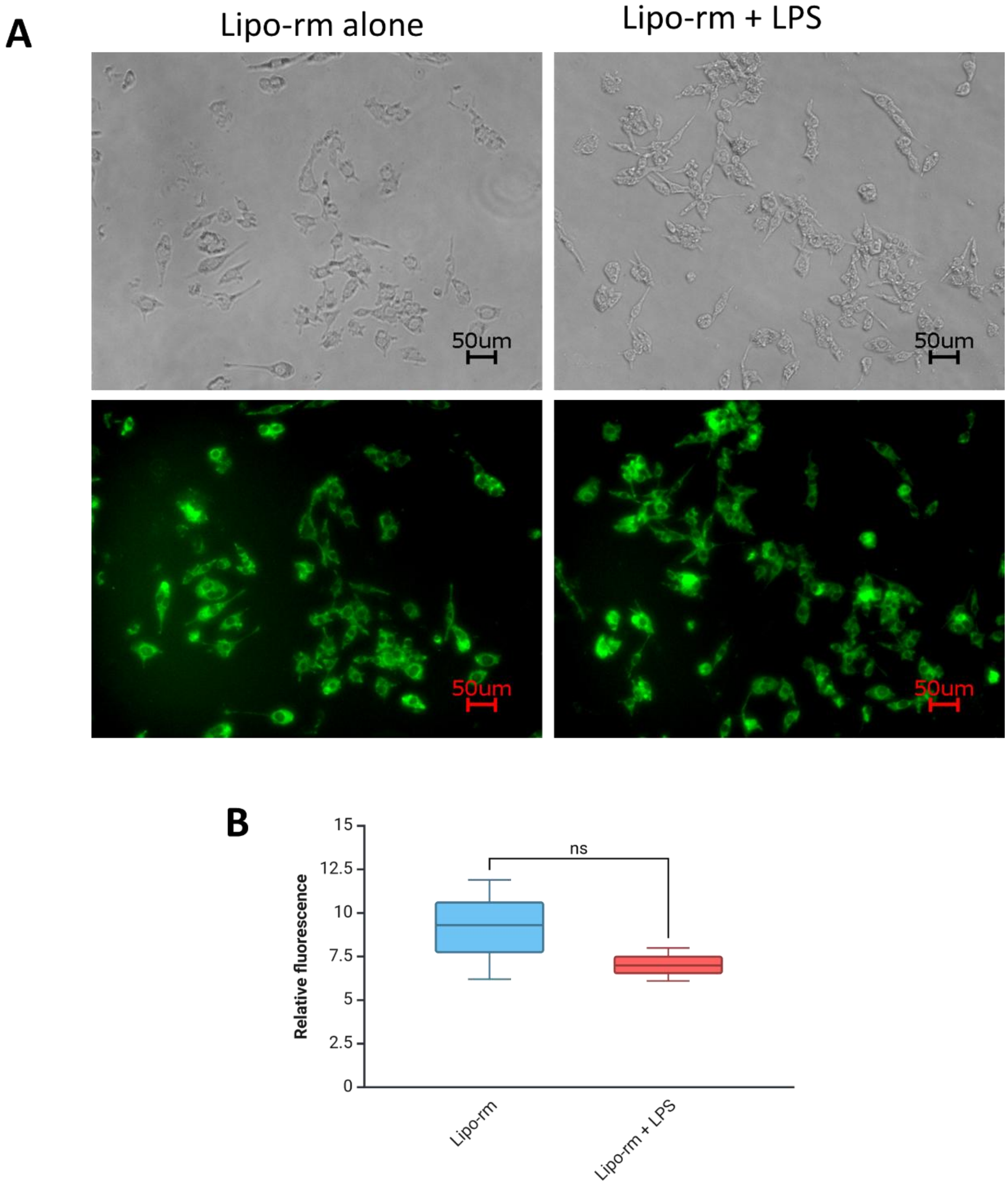

Fig. S9. Cellular uptake of Lipo-rm in LPS-pretreated RAW264.7 macrophage cells. (A) Representative fluorescence microscopy images showing Lipo-rm uptake in RAW264.7 macrophages in the absence (left) and presence (right) of LPS pretreatment. No significant difference in drug internalization was observed between the LPS-treated and untreated cells, indicating that LPS stimulation does not enhance or alter liposomal uptake in RAW264.7 cells (scale bar = 50 µM). **(B)** Quantitative analysis of rhodamine fluorescence intensity in RAW264.7 cells with and without LPS pretreatment, showing no statistically significant difference in cellular drug uptake. Data are presented as mean ± SD (n = 3); ns = not significant.

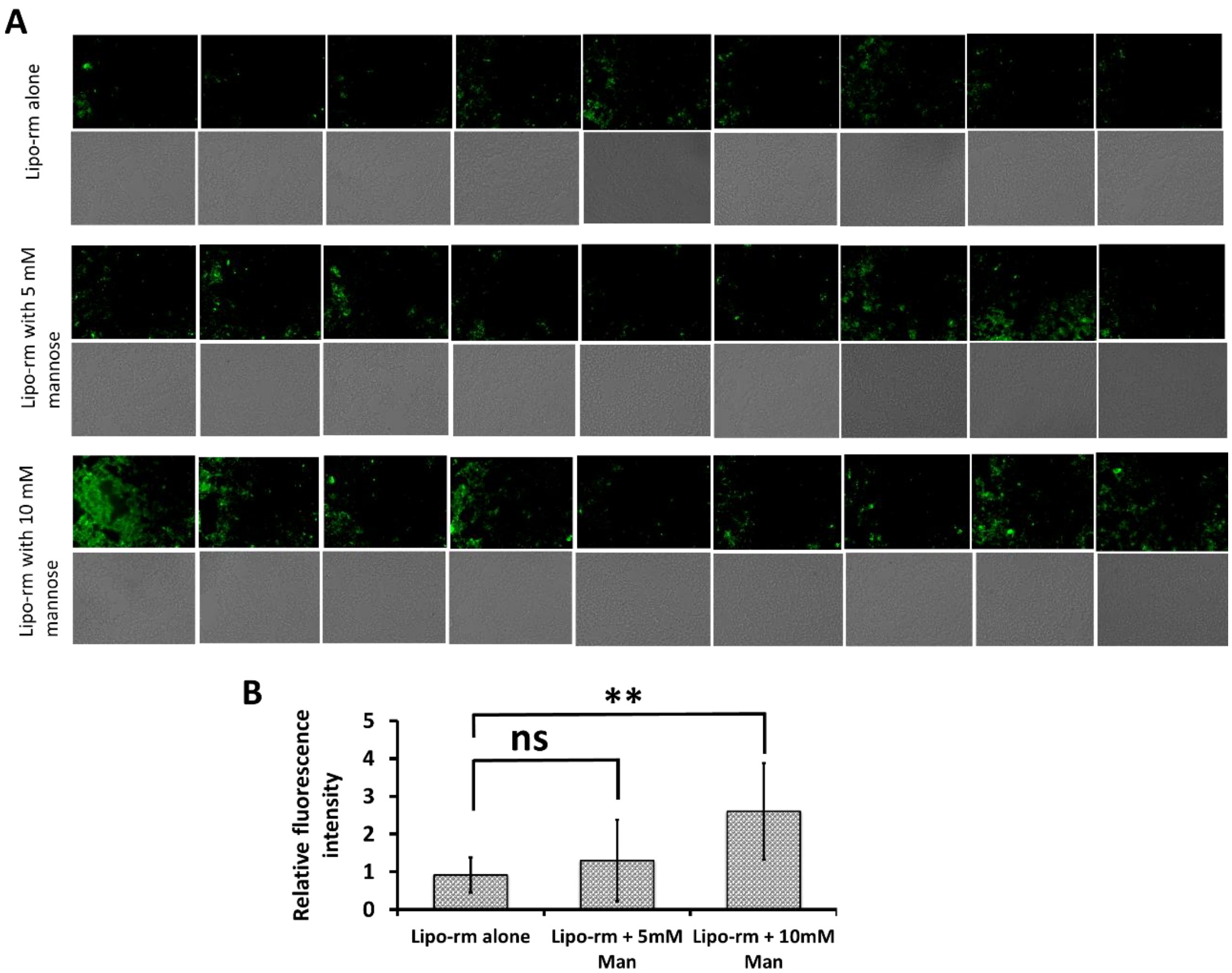

Fig. S10. Cellular uptake of Lipo-rm in the presence of mannose in RAW 264.7 macrophage cells. (A) Fluorescence microscopy images showing Lipo-rm uptake in RAW 264.7 cells with and without mannose pretreatment. The data indicate an enhanced cellular uptake of Lipo-rm following mannose exposure, suggesting mannose receptor-mediated endocytosis. Scale bar = 50 µm. **(B)** Quantitative analysis of rhodamine fluorescence intensity in RAW 264.7 cells following mannose pretreatment in a dose-dependent manner. The increased signal intensity confirms that mannose enhances Lipo-rm uptake. Data are presented as mean ± SD (n = 5). Statistical significance: *P < 0.05, **P < 0.01, ns = not significant.

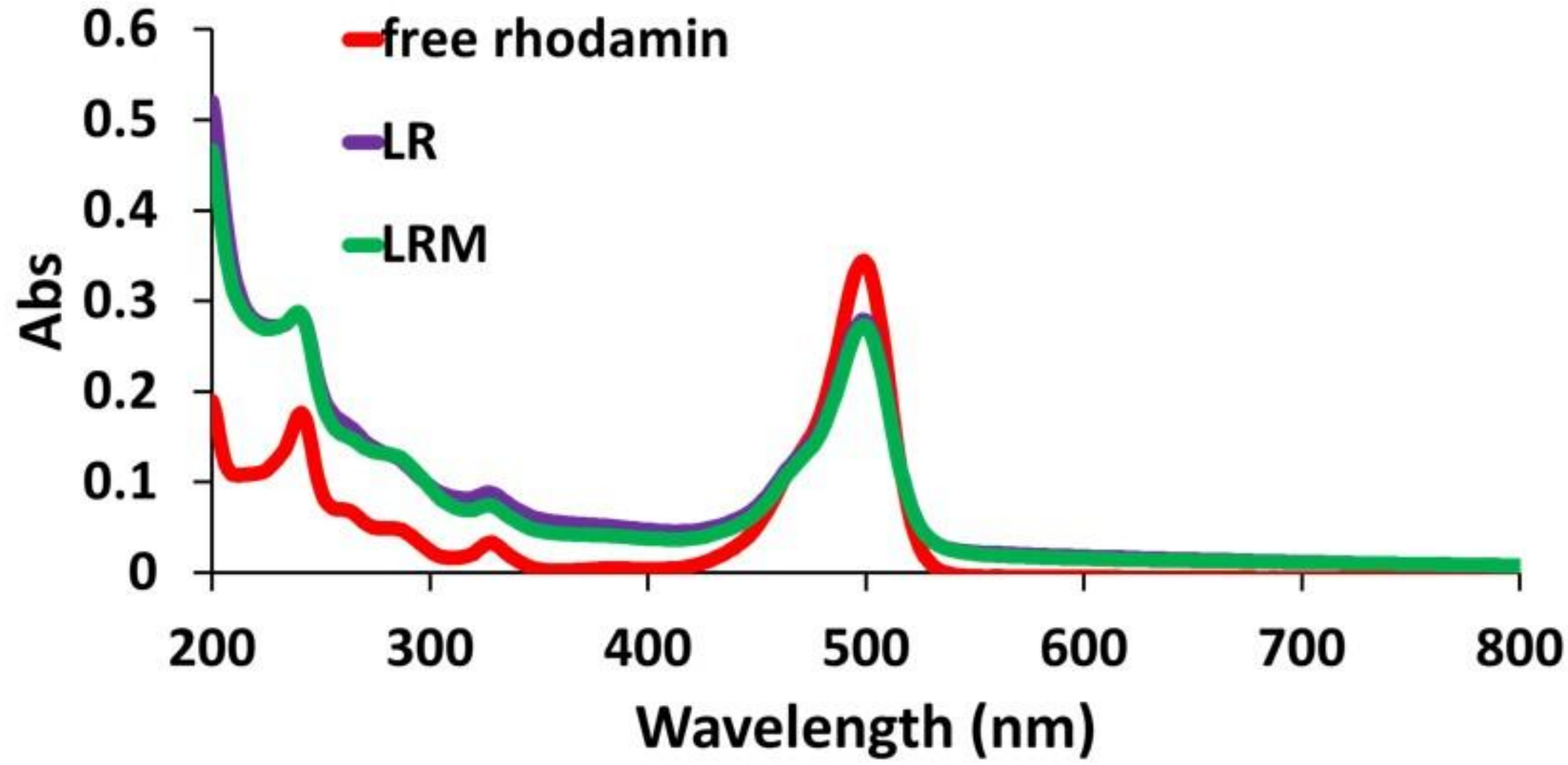

Fig. S11. Representative UV–vis spectra of rhodamine-loaded formulations in PBS at an equivalent dye concentration. All liposomal formulations and free rhodamine were adjusted to the same rhodamine concentration prior to cell treatment, as confirmed by overlapping absorbance spectra and comparable solution color intensity. These data verify that any differences in cellular responses between formulations are not attributable to variations in the administered rhodamine dose.

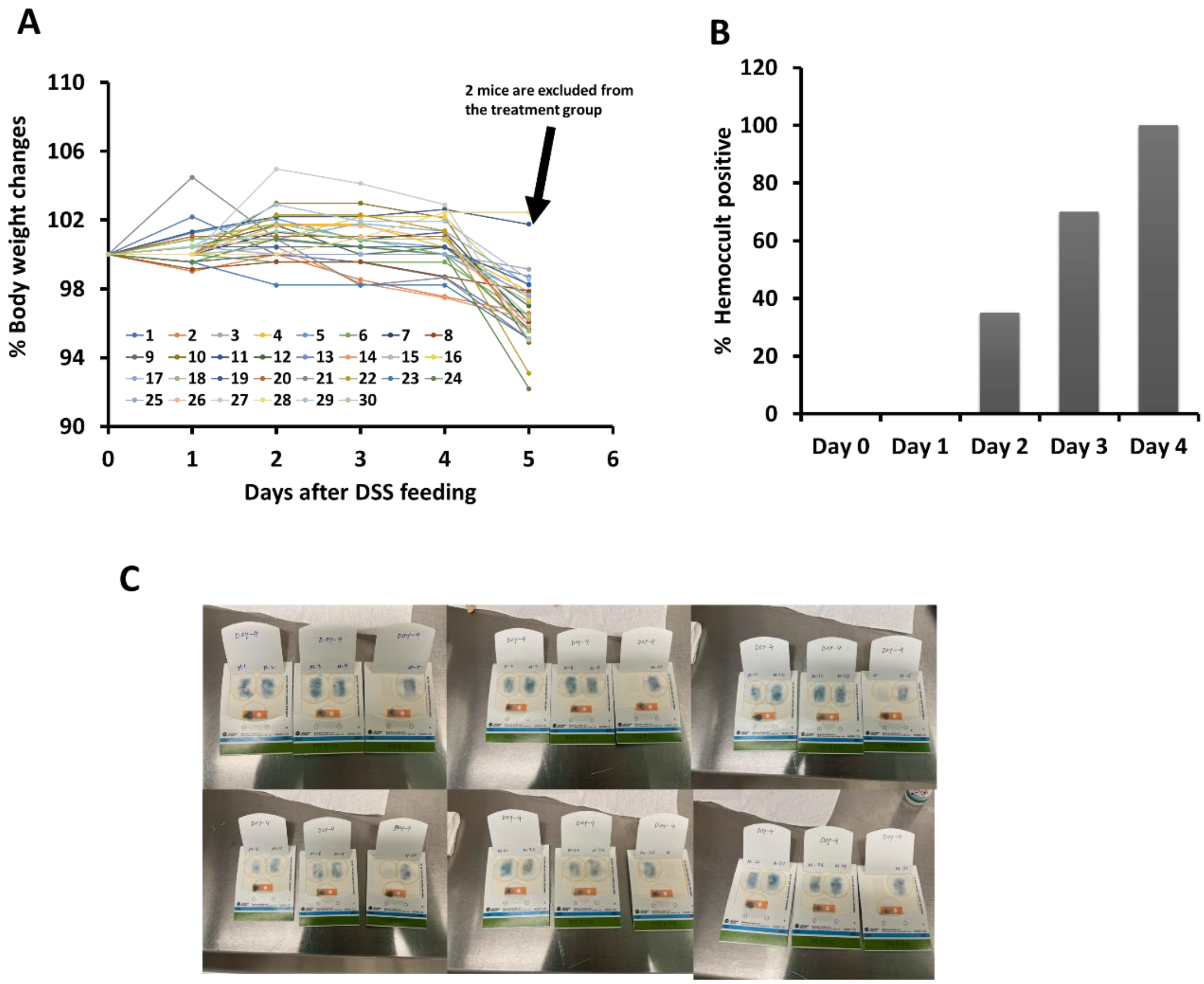

Fig. S12. Development of the 3.5% dextran sodium sulfate (DSS)-induced colitis model in C57BL/6 mice. (A) Body weight changes during DSS administration via drinking water. All mice exhibited significant weight loss by day 5, indicative of colitis progression. The black arrow denotes mice excluded from the treatment group due to the absence of weight loss. **(B)** Percentage of Hemoccult-positive mice following DSS administration. By day 4, all mice tested positive for occult blood in the stool, confirming the onset of colitis. **(C)** Representative Hemoccult test results from day 4 stool samples of DSS-treated mice. The appearance of a blue color indicates the presence of blood in the stool, confirming a Hemoccult-positive result.

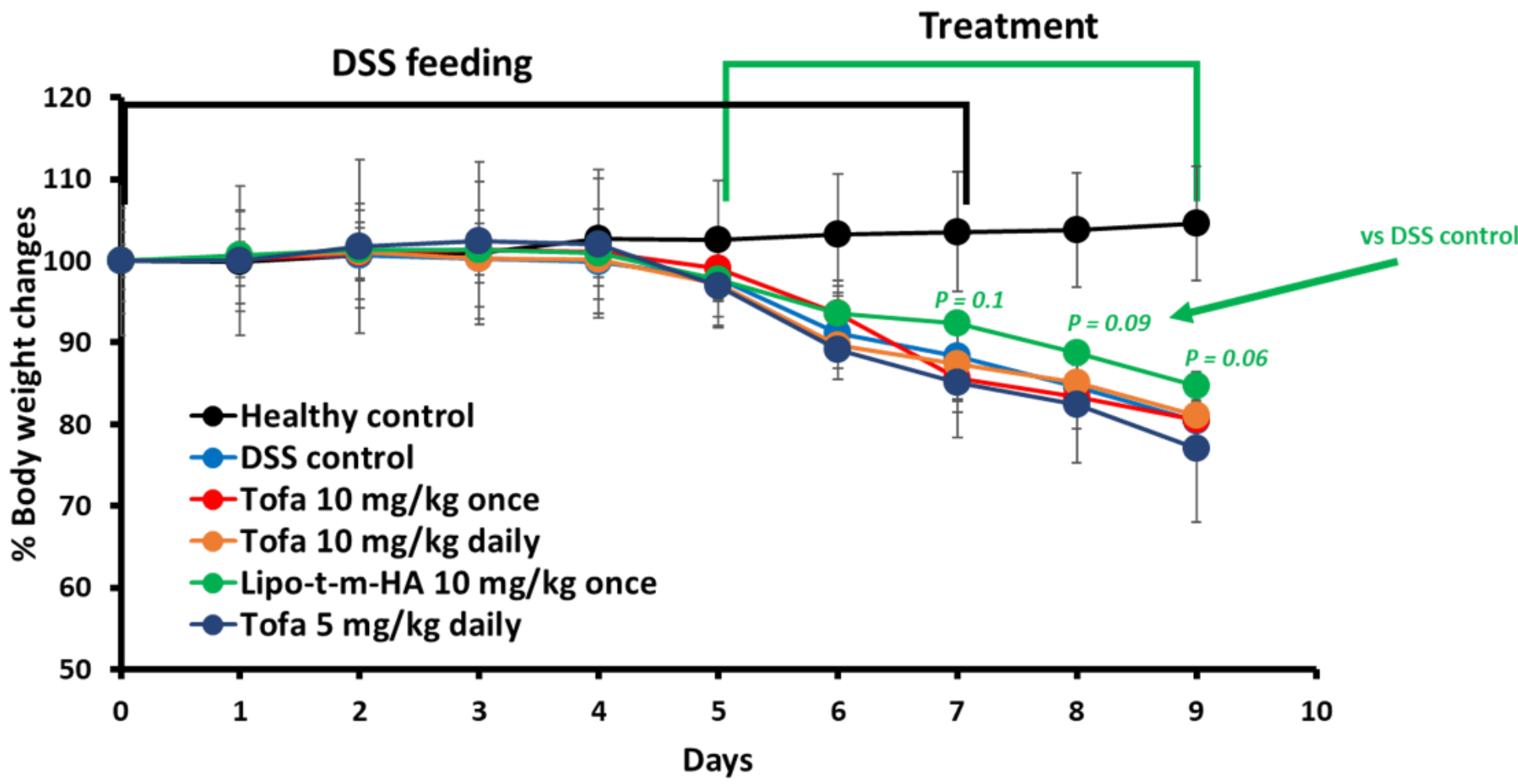

Fig. S13. Body weight changes in different treatment groups in DSS-induced colitis model. The green arrow indicates the p value between DSS control group and Lipo-tm-HA group.

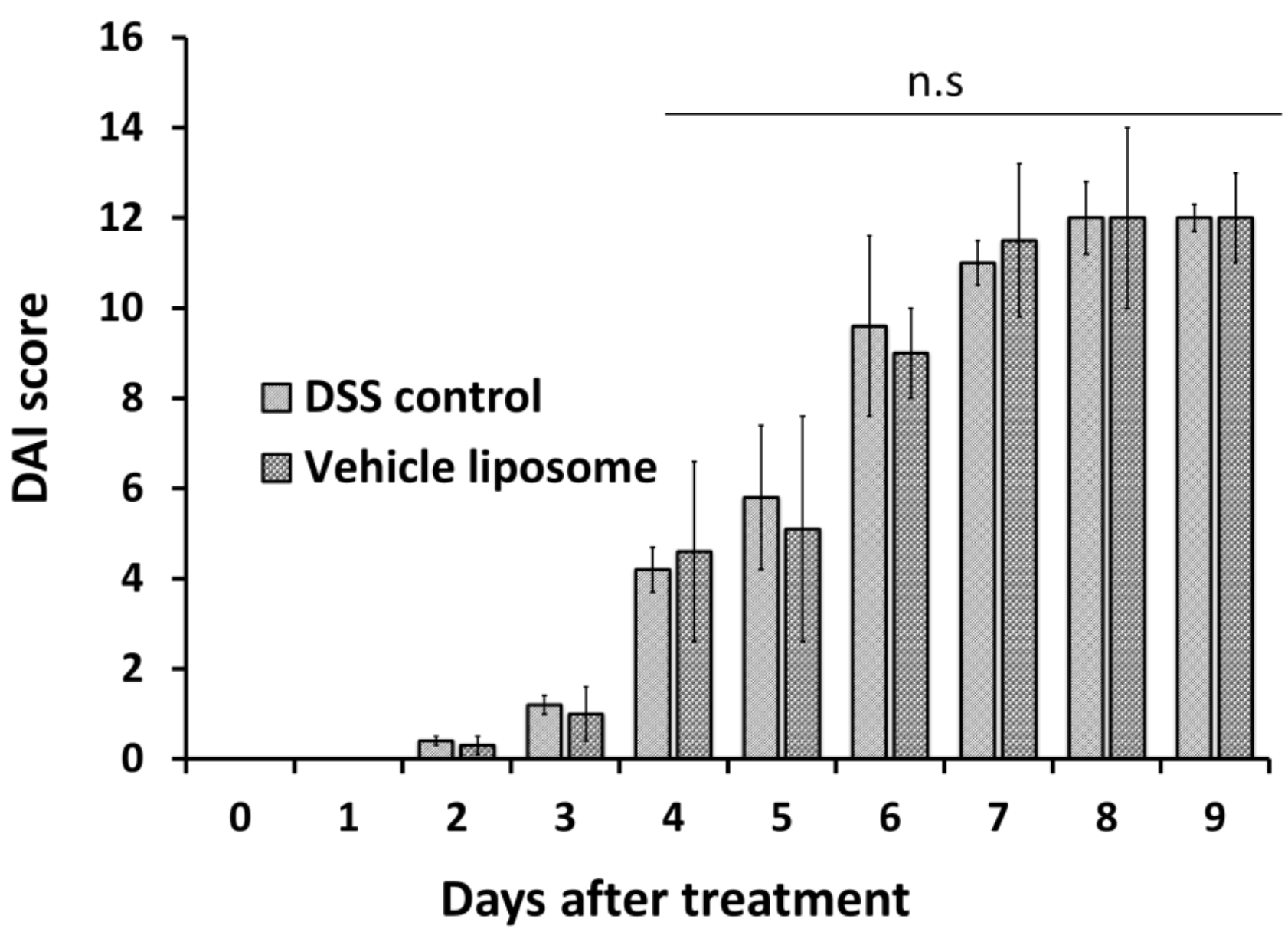

Fig. S14. Disease activity index (DAI) in DSS-induced colitis mice treated with vehicle liposomes (lacking tofacitinib). No significant improvement in DAI scores was observed in the vehicle-treated group compared to the DSS control group (±SD, n.s., not significant, n = 5). Data demonstrate that vehicle liposomes alone do not ameliorate colitis severity.

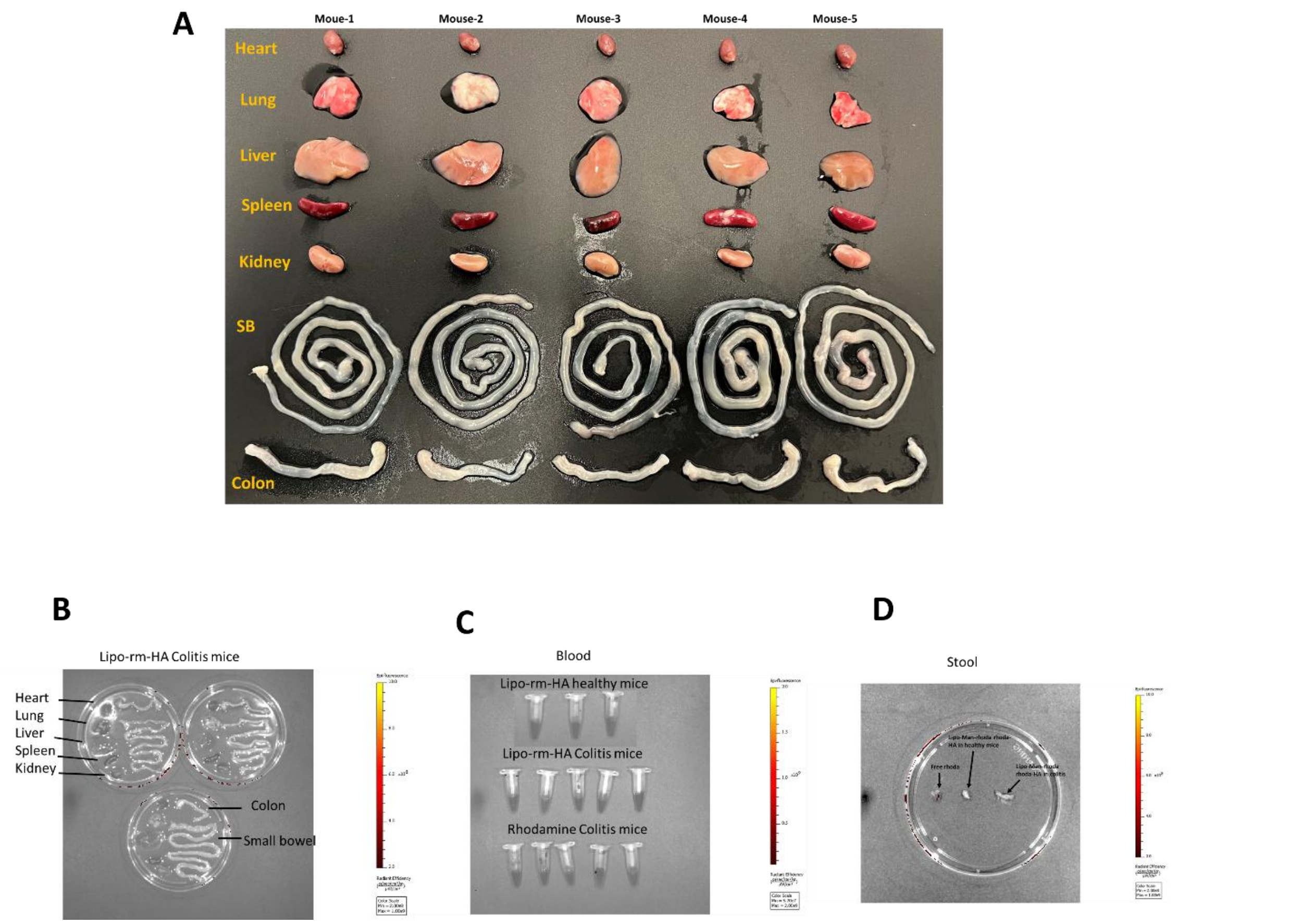

Fig. S15. Biodistribution of Lipo-rm-HA in C57BL/6 mice. (A) Ex vivo tissue images prior to fluorescence imaging. Mice were perfused with PBS, and the small intestine and colon were flushed with PBS to remove internal content. **(B)** Tissue distribution of Lipo-rm-HA (2.5 mg/kg, PO) in healthy, non-colitis mice visualized by in vivo imaging system (IVIS). No significant fluorescence signal was detected in the colon or other tissues, in contrast to DSS colitis mice, indicating the inflammation-targeting ability of Lipo-rm-HA. **(C)** Fluorescence quantification in blood after 24 hours of PO administration of free rhodamine and Lipo-rm-HA in both colitis and healthy mice. No significant fluorescence signal was detected across all groups, suggesting minimal systemic circulation. **(D)** Fluorescence imaging of stool collected 24 hours post-drug administration. No substantial fluorescence signal was detected by IVIS.

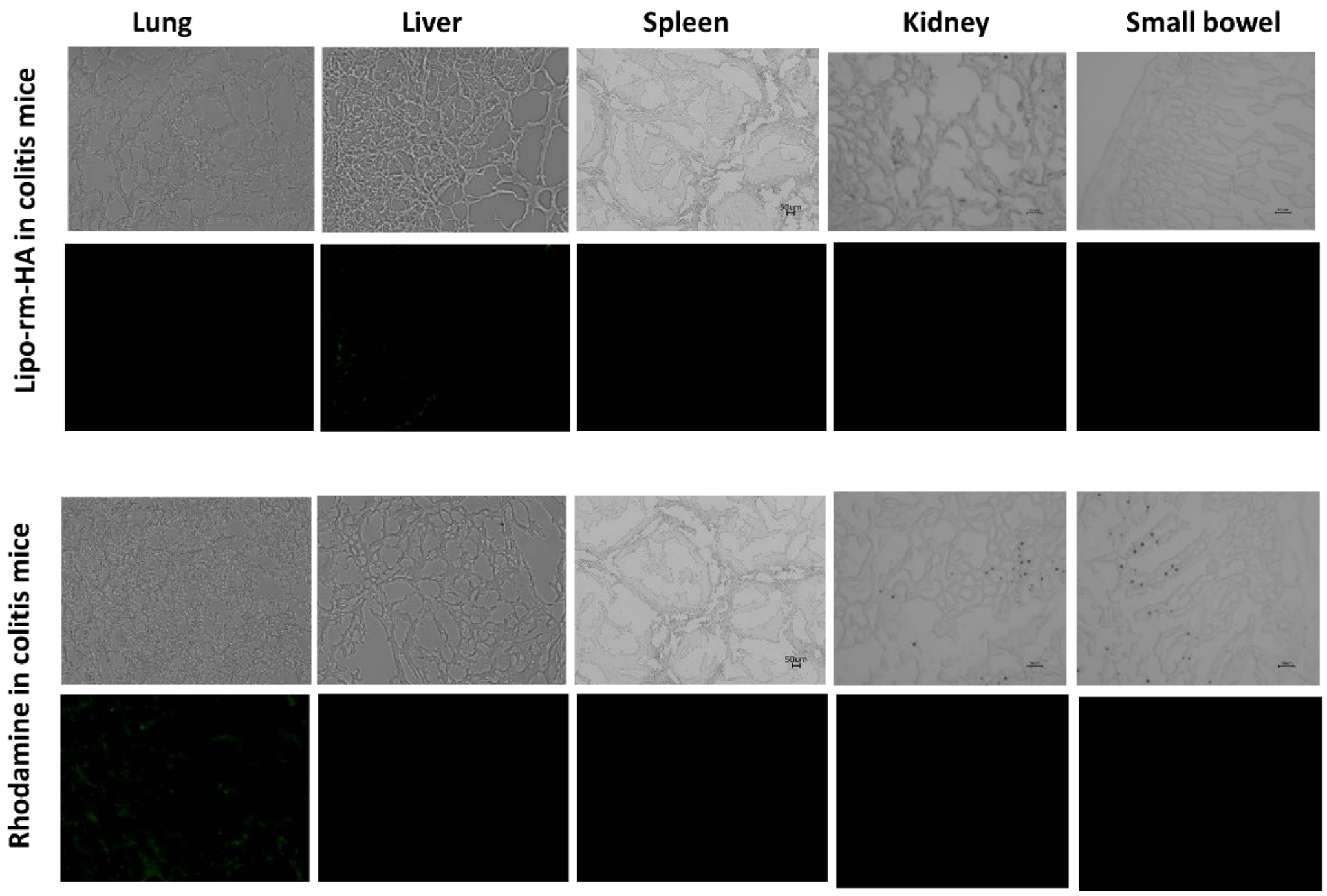

Fig. S16. Fluorescence microscopy images of different tissue sections from colitis mice. Colitis mice were treated with free dye rhodamine and Lipo-rm-HA (2.5 mg/kg, PO). After 24 hours, major tissues—including the lung, liver, spleen, kidney, and small intestine were collected. Tissues were fixed with 4% formalin, embedded in O.C.T. blocks, cryosectioned into 10 µm-thick slices, and mounted on slides for fluorescence imaging. Green fluorescence indicates the rhodamine signal, visualizing drug distribution and tissue localization. Scale bar: 50 µm.

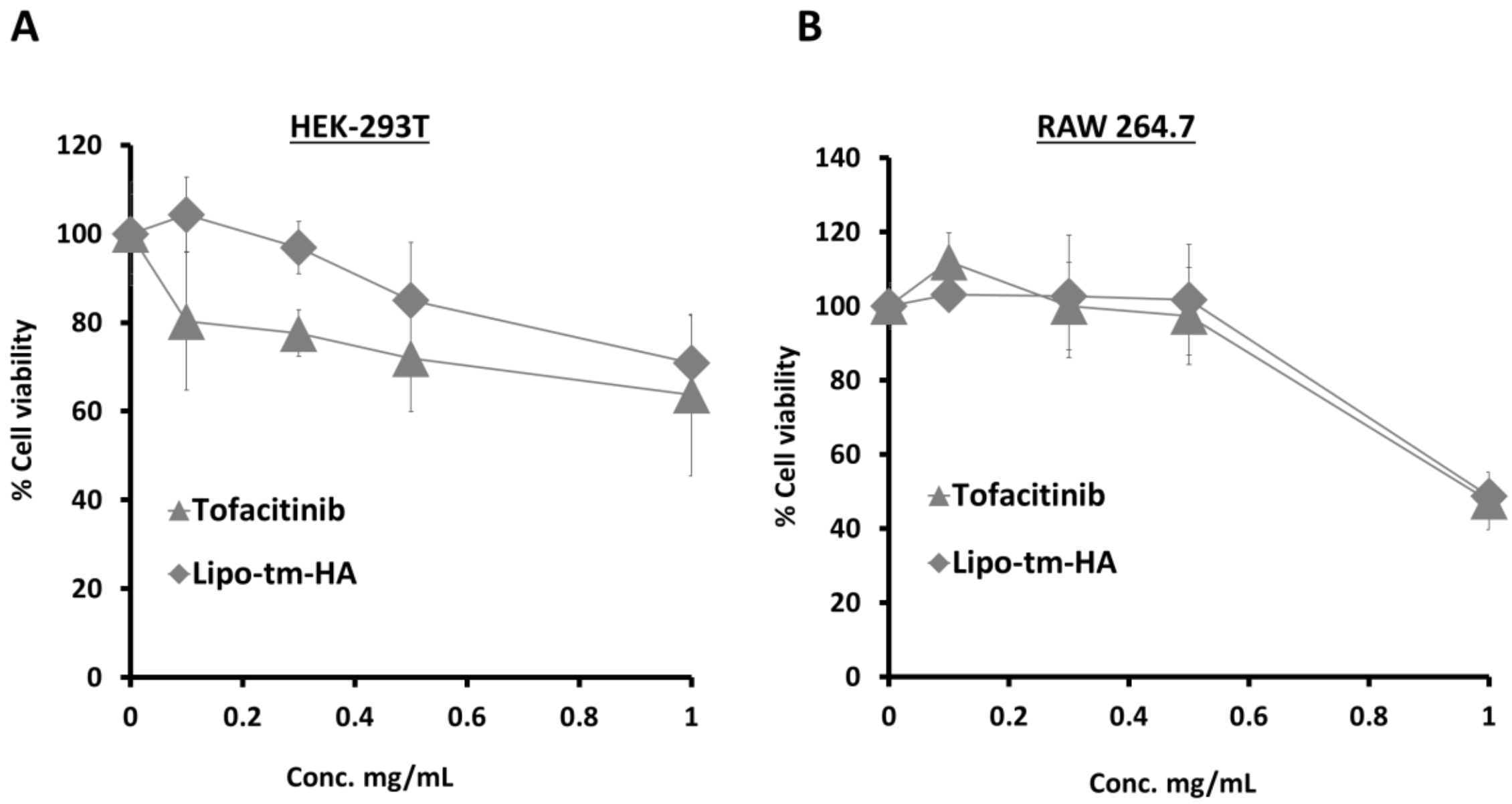

Fig. S17. Comparison of cytotoxicity between free tofacitinib and Lipo-tm-HA by MTT assay. (A) Cytotoxicity in human embryonic kidney (HEK293T) cells after 24 hours of drug treatment at 37°C. Briefly, HEK293T cells (1×10^3 cells/well) were cultured overnight, followed by treatment with free tofacitinib and Lipo-tm-HA at various concentrations. After 24 hours of incubation, cell viability was assessed using the MTT assay**. (B)** Cytotoxicity of tofacitinib and Lipo-tm-HA in macrophage RAW264.7 cells. Data are presented as mean ± SD (n = 5).
